# Evaluation of *bla*_OXA-48-like_ point mutation carbapenemase-producing *Enterobacterales* in Prapokklao Hospital, Thailand

**DOI:** 10.1128/spectrum.00198-24

**Published:** 2024-10-17

**Authors:** Sirijan Santajit, Witawat Tunyong, Thida Kong-Ngoen, Weewan Arsheewa, Woranich Hinthong, Pornpan Pumirat, Nitat Sookrung, Nitaya Indrawattana

**Affiliations:** 1 Department of Medical Technology, School of Allied Health Sciences, Walailak University, Nakhon Si, Thammarat, Thailand; 2 Research Center in Tropical Pathobiology, Walailak University, Nakhon Si, Thammarat, Thailand; 3 Department of Microbiology and Immunology, Faculty of Tropical Medicine, Mahidol University, Bangkok, Thailand; 4 Department of Microbiology, Prapokklao Hospital, Chanthaburi, Thailand; 5 Princess Srisavangavadhana College of Medicine, Chulabhorn Royal Academy, Bangkok, Thailand; 6 Department of Infection Biology, Faculty of Infectious and Tropical Diseases, London School of Hygiene and Tropical Medicine, London, United Kingdom; 7 Siriraj Center of Research Excellence in Allergy and Immunology, Faculty of Medicine Siriraj Hospital, Mahidol University, Bangkok, Thailand; 8 Biomedical Research Incubator Unit, Research Department, Faculty of Medicine Siriraj Hospital, Bangkok, Thailand.; University of Pretoria, Pretoria, Gauteng, South Africa

**Keywords:** antibiotic resistance, *bla*_OXA-48-like_, CPE, carbapenemases, eCIM, modified hodge test, mCIM

## Abstract

Carbapenemase-producing *Enterobacterales* (CPE) isolates increasingly carry oxacillinase-48 (OXA-48)-like enzymes encoded by *bla*_OXA-48-like_, which can confer high levels of carbapenem resistance. This aims to determine the prevalence of CPE and genetic variation among *bla*_OXA-48-like_-carrying isolates recovered from Prapokklao Hospital in Chanthaburi Province, Thailand in 2016–2017. In total, 122 carbapenem-resistant *Enterobacterales* (CRE) isolates were recovered from clinical samples. CRE were evaluated using standard biochemical tests and MIC test strips. Carbapenemase production was assessed through the modified Hodge test (MHT), modified carbapenem inactivation method (mCIM), and EDTA-modified carbapenem inactivation method (eCIM). Detection of *bla*_OXA-48-like_ mutations was conducted via PCR and confirmed by Sanger sequencing. Among these CRE isolates, 72 (59.02%), 44 (36.07%), 3 (2.46%), and 3 (2.46%) were *Klebsiella pneumoniae*, *Escherichia coli*, *Enterobacter aerogenes*, and *Enterobacter cloacae*, respectively. The MHT identified carbapenemase production in 108 isolates (88.52%). Based on the mCIM, 81 isolates (66.39%) were carbapenemase producers. Seventy-three isolates (59.84%) were eCIM-positive, indicating metallo-β-lactamase production. Three distinct genetic variants of the *bla*_OXA-48-like_ gene were identified among the isolates, including the wild-type and two point mutation types harboring the mutations E168Q and S171A (mutation type 1) and E168Q, S171A, and R214S (mutation type 2). Multiple-sequence alignment and *in silico* analysis revealed variation of R214 located in the β5–β6 loop. This study identified *bla*_OXA-48-like_ point mutation groups and carbapenemase production, predominantly metallo-β-lactamases, among CRE isolates, especially *K. pneumoniae* and *E. coli*. These findings highlight the importance of implementing stringent infection control measures and active antimicrobial resistance surveillance to combat the spread of difficult-to-treat, metallo-β-lactamase-producing CRE in healthcare settings.

**IMPORTANCE:**

In this study, we aimed to investigate genetic variation and CPE among bla_OXA-48-like_ carrying isolates recovered from Prapokklao Hospital, Chanthaburi Province, Thailand, during 2016–2017. A total of 122 carbapenem-resistant Enterobacterales (CRE) were recovered from clinical samples in Prapokklao Hospital. All CRE samples were confirmed by standard biochemical tests and minimum inhibitory concentration (MIC) test strips (E-test). The carbapenemase production was determined using the modified Hodge test (MHT), the modified carbapenem inactivation method (mCIM), and EDTA-CIM (eCIM). Three single mutations (E168Q, S171A, and R214S) were characterized in this study. This mutation might reflect the hydrolysis of the modified β-lactam spectrum, especially carbapenem, by OXA-48-like. Our report provides evidence of the bla_OXA-48-like_ point mutation and carbapenemase-producing phenotype of CRE detected in this healthcare setting. Effective control measures and active surveillance of drug resistance in nosocomial pathogens are crucial for controlling diseases associated with difficult-to-treat bacteria.

## INTRODUCTION

The global spread of carbapenemase-producing *Enterobacterales* (CPE) has contributed to the increased prevalence of carbapenem resistance in recent decades, representing a danger to public health (1). Carbapenem-resistant *Enterobacterales* (CRE) comprises a substantial order of Gram-negative bacteria that are non-susceptible (intermediate or resistant) to at least one carbapenem (ertapenem, meropenem, doripenem, or imipenem) (2). The most concerning carbapenem-resistant organisms (CROs) include *Escherichia coli*, *Klebsiella pneumoniae*, and *Enterobacter* spp (3). These bacteria can cause serious conditions such as urinary tract infections, sepsis, diarrhea, and systemic infections. Drug-resistant strains of these organisms have been documented in multiple regions (4, 5). CRE isolates are particularly alarming because of their involvement in various infections that result in elevated mortality rates and their potential to disseminate carbapenem resistance through mobile genetic elements (4, 6). Strains carrying plasmid-encoded resistance genes efficiently transfer these genes to other species.

The emergence of carbapenem resistance among *Enterobacterales* primarily arises from the production of carbapenem-hydrolyzing β-lactamases, specifically carbapenemases belonging to classes A (KPC), B [metallo-β-lactamases (MBLs); e.g., New Delhi metallo-β-lactamase (NDM), Verona integron‐encoded metallo-β-lactamases (VIM), and Imipenemase (IMP)], and D [oxacillinase-48 (OXA-48)-like and its variants] (7). Class A and D enzymes employ a hydrolytic mechanism centered around serine, whereas class B enzymes are MBLs characterized by the presence of zinc in their active sites (8). Currently, more than 1,000 β-lactamase variants have been documented, and their abundance continues to steadily increase (9, 10). Since the early 2000s, there have been global reports of CPE outbreaks, and CPE has become established in some countries (11). In 2018, data submitted to the European Antimicrobial Resistance Surveillance Network revealed that 0.1% of *E. coli* isolates and 7.5% of *K. pneumoniae* isolates from invasive infections were resistant to carbapenem (12). In Europe, the incidence of CPE, including both infections and colonization, has gradually increased since 2009. Although most cases involve patients with recent travel or hospitalization abroad, there is a growing number of locally acquired cases, often linked to OXA-48-like CPE (13, 14).

The key mechanism of resistance to CRE is the acquisition of carbapenemases, a group of β-lactamases. These enzymes can be classified into classes A (e.g., KPC), B [metallo-β-lactamases (MBLs); e.g., NDM, VIM, IMP), and D (OXA-48-like and variants). Class D carbapenemases, such as OXA-48-like, play a significant role in the emergence of resistance to last-resort β-lactam antibiotics. OXA-48-like β-lactamase confers resistance to carbapenems and can lead to life-threatening infections due to limited treatment options. Class D carbapenemases play a significant role in the emergence of resistance to β-lactam antibiotics, especially those regarded as treatment options of last resort (15). A critical clinically significant carbapenemase is the class D β-lactamase OXA-48-like, which poses a considerable threat to public health because it confers resistance to the last-line antibiotics carbapenems, resulting in a range of life-threatening infections and their rapid global dissemination (16, 17).

OXA-48-like β-lactamase was discovered in 2004 in a *K. pneumoniae* isolate from Istanbul, Turkey. Subsequently, it spread quickly across Europe, the Middle East, and the Mediterranean region, posing a growing threat (18). Several OXA-48-like variations have been reported globally since OXA-48-like was first identified (16, 19). Although OXA-48-like effectively hydrolyzes penicillins, its ability to hydrolyze carbapenems is limited, and it exhibits minimal activity against extended-spectrum cephalosporins. The recent identification of several OXA-48-like variants indicates their wide distribution. A four-amino acid deletion in the β5–β6 loop exhibits expanded-spectrum cephalosporin hydrolytic activity but no ability to hydrolyze carbapenem (20). However, when combined with other resistance mechanisms, such as compromised outer membrane permeability and extended-spectrum β-lactamase (ESBL) production, the resistance level to these antibiotics in OXA-48-like-producing bacteria can be significantly higher. This situation can be highly lethal because treatment options for serious infections caused by these bacteria are severely restricted (21). For infection control and prevention, a CPE screening test should be performed (22, 23). According to a European study, intrahospital and interhospital transmission of CPE occurs more frequently within nations than between nations (24). To halt the spread of CPE, hospital-level and nationwide surveillance is required.

Although OXA-48-like variants have been reported globally, there is a scarcity of information regarding the sequence types disseminated in Thailand. This study aimed to examine the prevalence of CPE and *bla*_OXA-48-like_ variant genes/point mutations at Prapokklao Hospital in Chanthaburi Province, Thailand. Understanding the local epidemiology and molecular characteristics of CPE is crucial for implementing effective infection control measures and antimicrobial resistance surveillance to combat the spread of these difficult-to-treat pathogens in healthcare settings.

## MATERIALS AND METHODS

### CRE study design and bacterial confirmation

Carbapenem-resistant *Enterobacterales* (CRE) was defined as isolates non-susceptible (intermediate or resistant) to at least one carbapenem antibiotic (meropenem or ertapenem) based on minimum inhibitory concentrations (MICs) determined by E-test according to the manufacturer’s instructions (Liofilchem, Roseto degli Abruzzi, Italy) (1, 2, 22).

This cross-sectional study estimated the *bla*_OXA-48-like_ gene among bacterial isolates recovered at Prapokklao Hospital between 2016 and 2017.

**Fig 1:**
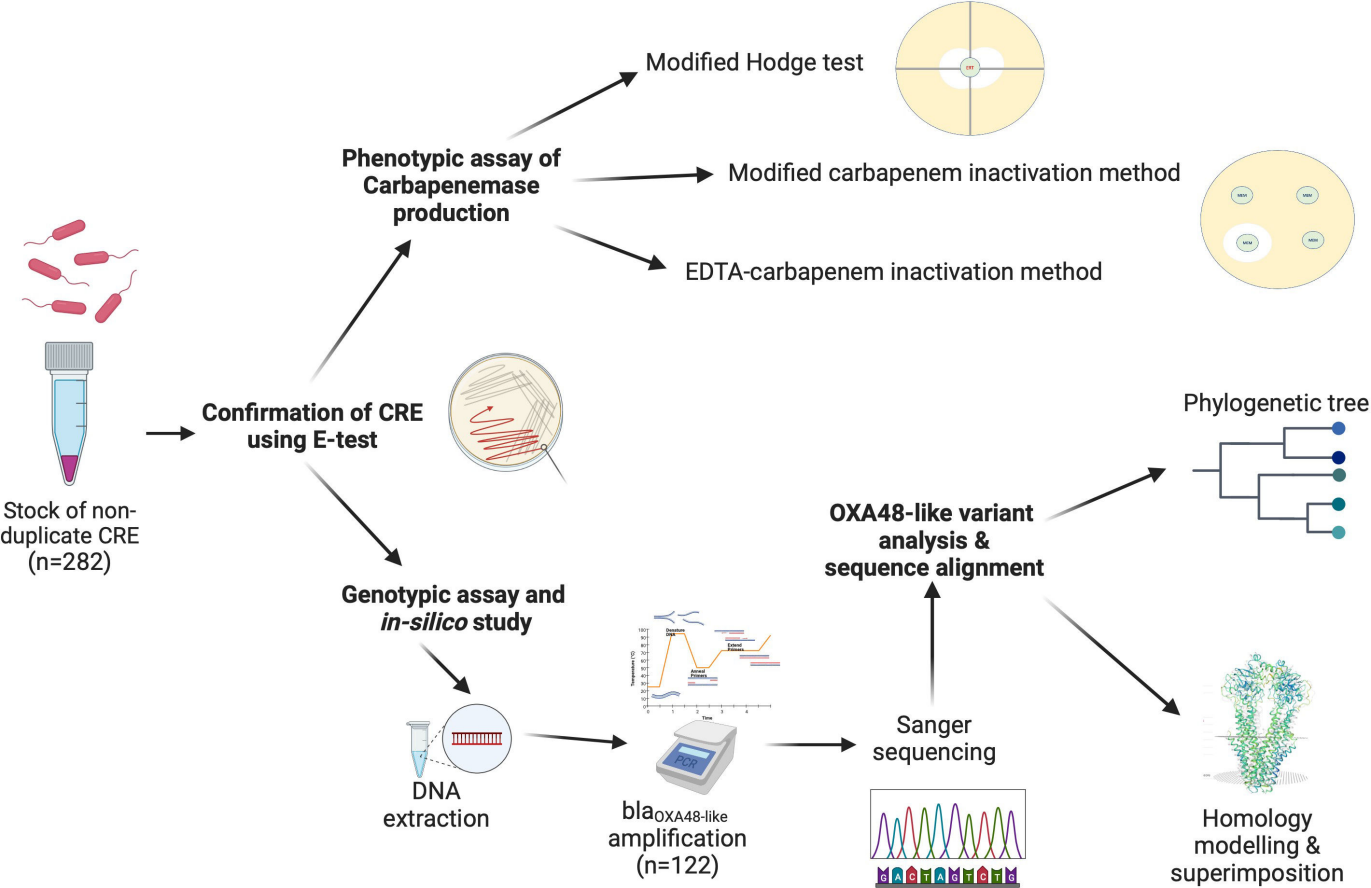
Graphical abstract of the study. Bacterial culture and confirmation of CRE from the stock solutions. Phenotypic assays for carbapenemase production and the detection of OXA-48-like point mutations were performed.

A total of 282 non-duplicate CRE isolates were identified by colony morphology, Gram staining, and biochemical tests including oxidase, triple sugar iron utilization, ornithine decarboxylase, indole production, motility, and citrate utilization tests (12). The *bla*_OXA-48-like_ gene was amplified and further subjected to DNA sequencing to investigate sequence mutations, and analyzed *in silico* (20). Carbapenemase production was investigated in *bla*_OXA-48-like_-positive samples. A graphical abstract summarizing this study is presented in Fig. 1.

### Detection of *bla*_OXA-48-like_, DNA sequencing, and multiple sequence alignment

Bacterial genomic DNA was isolated from bacteria grown overnight at 37°C in 3  mL of Luria–Bertani (LB) broth. Bacterial cultures were centrifuged for 1 min at 12,000 rpm, and the supernatant was removed. Crude DNA extracts were obtained by suspending the pellet in 300 µL of distilled water and boiling at 95°C for 10 min, followed by centrifugation at 12,000  rpm for 5 min. The supernatant containing DNA was transferred to a new Eppendorf tube, and the DNA quantity was measured using a NanoDrop-1000 spectrophotometer (Thermo Fisher Scientific, Waltham, MA, USA).

PCR targeting the *bla*_OXA-48-like_ gene was used as the standard to assess the performance of genotypic tests. Therefore, PCR amplification to detect *bla*_OXA-48-like_ was performed on a PCR cycler (Bio-Rad, Hercules, CA, USA). The sequence containing the β5–β6 loop, which comprises the catalytic part of the OXA-48-like enzyme against β-lactams, was amplified using the specific primer pair OXA-48-F (forward primer; 5′-TTGGTGGCATCGATTATCGG-3′) and OXA-48-R (reverse primer; 5′-GAGCACTTCTTTTGTGATGGC-3′) (25). The amplification mixture (25 µL) contained *Taq* DNA polymerase, 10 × *Taq* buffer, 25 mM MgCl_2_, 10 mM dNTPs, 10 mM forward and reverse primer solutions, 1.25 units/L (Thermo Fisher Scientific), and 100 ng/L DNA template. PCR was performed with initial denaturation at 95°C for 5 min, followed by 35 cycles of denaturation at 95°C for 30 s, annealing at 56°C for 30 s, and extension at 72°C for 30 s, with a final extension at 72°C for 7 min. The PCR products were analyzed by electrophoresis on 1.5% agarose gels in 0.5 × Tris-borate-EDTA buffer. The gels were stained with SafeView FireRed (Applied Biological Materials, Richmond, BC, Canada), and the PCR products were visualized with UV light using the ChemiDoc MP imaging system (Bio-Rad).

Clinical *K. pneumonia* isolates harboring *bla*_OXA-48-like_ from a previous study were used as positive controls (26). The *bla*_OXA-48-like_ amplicons (743 bp) were purified and subjected to Sanger sequencing. The nucleotide sequences were examined using software available on the National Center for Biotechnology Information website (http://www.ncbi.nlm.nih.gov). Multiple sequence alignments were generated using CLC Sequence Viewer Version 8 (27). The phylogenetic tree of the *bla*_OXA-48-like_ variant/point mutations was constructed using MEGA11 software (28) and the neighbor-joining method (29).

### Modified Hodge test

The MHT was performed on CRE isolates according to the (30) guidelines to detect the presence of carbapenemase (30). The following medium and carbapenem antibiotics were used: 10 µg ertapenem disks (Oxoid, Thermo Fisher Scientific), 10 µg meropenem disks (Oxoid), and Mueller–Hinton agar (MHA; Difco, Detroit, MI, USA). A 0.5 McFarland standard suspension of *E. coli* ATCC 25922, the MHT carbapenem susceptible indicator organism, was suspended in Mueller–Hinton broth (Difco) after being grown overnight.

An undiluted suspension or a 1:10 diluted suspension was plated onto MHA in accordance with the CLSI disk diffusion procedure (30). Each of the ertapenem or meropenem disks was placed in the middle of the plate, and the test isolates were thoroughly streaked using a 10 µL disposable loop from the edge of the central disk to the plate periphery. Each plate was tested with up to four isolates. After overnight incubation at 35°C in ambient air, the indicator organism inhibition zone was carefully observed for enhanced growth around the test organism streak (a cloverleaf-type indentation) that appeared after the test organism grew at the intersection of the streak. Two indentation levels, measuring 2 and 3 mm, were employed as positive cutoff values. The enhanced growth indicates positive carbapenemase production, whereas no enhanced growth indicates negative carbapenemase production.

### Modified carbapenem inactivation method and EDTA-modified carbapenem inactivation method

The goal of the mCIM and eCIM is to assess the potential of the tested bacteria to produce carbapenemases. The mCIM and eCIM involve incubating the bacteria with a meropenem disk and subsequently assessing whether the antibiotic is inactivated. The use of EDTA in the eCIM method enhances the detection of MBLs, which comprise a class of carbapenemases that are inhibited by chelating agents such as EDTA. Briefly, a 1 µL loopful of bacteria was resuspended in a 2-mL tube containing tryptic soy broth (TSB). Then, in a separate tube containing 2 mL of TSB supplemented with 5 mM EDTA (achieved by adding 20 µL of 0.5 mM EDTA to 2 mL of TSB), another 1 µL loopful of bacteria was resuspended. Then, a meropenem disk was placed in each tube, and these tubes were incubated at 35°C for 4 h. After this incubation period, the disks were placed on MHA plates that had just been inoculated with a 0.5 McFarland suspension of *E. coli* ATCC 25922 strains that were susceptible to carbapenem.

The reaction plates were incubated at 35°C for 20 h, and the results of the mCIM and eCIM were interpreted in accordance with the CLSI 2020 (30) criteria. The mCIM results were interpreted as positive if the zone was 6–15 in size, intermediate (defined as positive) when pinpoint colonies were found inside a 16–18 mm zone, and negative if the zone was 19 mm or larger. If the eCIM zone size was at least 5 mm larger than that measured in the mCIM, the isolate was indicated as positive for metallo-carbapenemase production, whereas if the increase in zone size was less than 4 mm, the isolate was classified as negative. *E. coli* ATCC 25922 (carbapenemase positive) and *K. pneumoniae* strains (carbapenemase negative, *bla*_KPC_ positive, and *bla*_NDM_ positive) from a previous study (26) were used as internal controls for the mCIM and eCIM tests in compliance with CLSI criteria. In addition, the MHT, mCIM, and eCIM were independently repeated by two different investigators to determine the repeatability of the results.

### *In silico* studies: homology modeling and superimposition

Mutations within the OXA-48-like enzymes of representative CPE isolates were first identified by sequencing, followed by sequence alignment with reference sequence (accession number NG_049762.1:101–898) to determine the specific single nucleotide polymorphisms (SNPs). The roles of these mutations were then established using predictive homology modeling-based 3D structure analysis and protein superimposition of carbapenems within the binding pocket (β5–β6 loop). Structural investigation of OXA-48-like wild-type (WT) and mutation groups was performed using SWISS-MODEL (http://swissmodel.expasy.org) (31). The quality of the generated homology models of OXA-48-like enzymes was then evaluated using Ramachandran plots (32). Superimposition to compare OXA-48-like mutation groups with the WT complexed with meropenem (PDB ID: 6ZRP) was performed using PyMol software (PyMol Molecular Graphics System, Version 2 edu, Schrodinger, LLC) (33). The CLICK server (http://mspc.bii.a-star.edu.sg/click), which is a topology-independent tool, compared the superimposed 3D structures without a scoring function measuring structural similarity (34). Discovery Studio 3.5 was used to analyze and visualize all models.

## RESULTS

### Phenotypic detection of CPE

**Fig 2:**
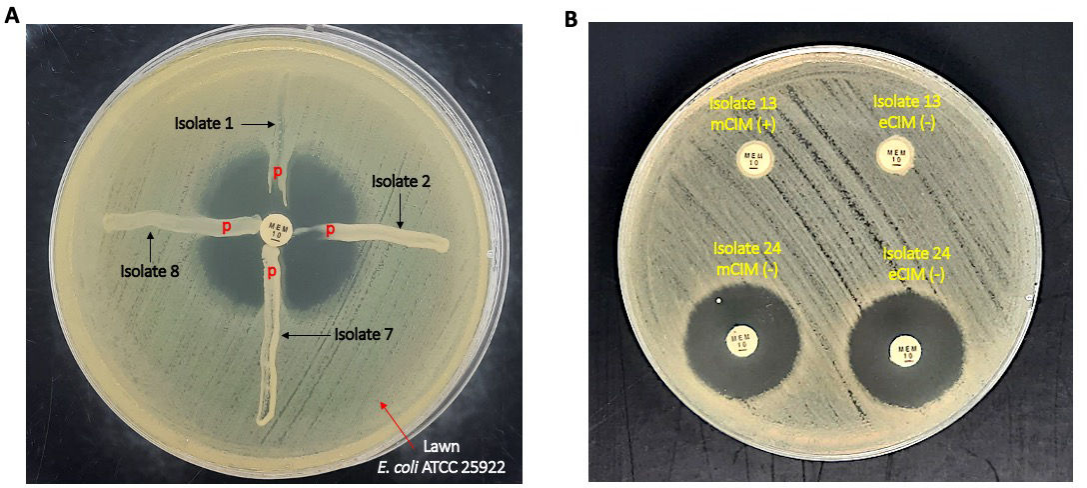
Screening and identification of carbapenemase-producing strains using the MHT, mCIM, and eCIM. (**A**) Representative results of the MHT. Isolates 1, 2, 7, and 8 were MHT positive. (**B**) Representative results of the mCIM and eCIM. Thirteen isolates were mCIM positive and eCIM negative, whereas 24 isolates were mCIM negative and eCIM negative. n, negative; p, positive.

The phenotypic analysis employed the MHT, mCIM, and eCIM to investigate carbapenemase-producing strains according to the CLSI guidelines for 2020. Out of 122 isolates, 108 (88.52%) CRE isolates were MHT positive, including 62 *K*. *pneumoniae* isolates (50.82%), 41 *E. coli* isolates (33.61%), 3 *E. aerogenes* isolates (2.46%), and 2 *E. cloacae* isolates (1.64%). The mCIM and eCIM results indicated that 93 isolates produced carbapenemase (76.23%). Of these, 23 isolates produced serine carbapenemase (18.85%), including 3 *E. coli* isolates (2.46%) and 20 *K*. *pneumoniae* isolates (16.39%). In all, 70 isolates (81.15%) were metallo-β-lactamases producers, including 36 *E. coli* isolates (29.51%), 29 *K*. *pneumoniae* isolates (23.77%), 3 *E. aerogenes* isolates (2.46%), and 2 *E. cloacae* isolates (1.64%, Table 1). A representative result is presented in Fig. 2.

**TABLE 1:** Phenotypic detection of CRE using the MHT, mCIM, and eCIM and evaluation of OXA-48-like mutation groups^*a*^

Bacterial pathogens	Carbapenemase production						Evaluation of OXA-48-like mutation groups		
	MHT				mCIM	eCIM	No. of OXA-48-like type mutations (%)		
	Ertapenem		Meropenem						
	No. of negative samples	No. of positive samples	No. of negative samples	No. of positive samples	No. of serine carbapenemase positive	No. of MBLs positive	E168Q	S171A	R214S
									
*E. coli* (*n* = 44)	11 (9.02%)	33 (27.05%)	3 (2.46%)	41 (33.61%)	3 (2.46%)	36 (29.51%)	41 (33.61%)	41 (33.61%)	34 (27.87%)
*K. pneumoniae* (*n* = 72)	11 (9.02%)	61 (50.00%)	10 (8.20%)	62 (50.82%)	20 (16.39%)	29 (23.77%)	68 (55.74%)	68 (55.74%)	67 (54.92%)
*E. aerogenes* (*n* = 3)	0 (0%)	3 (2.46%)	0 (0%)	3 (2.46%)	0 (0%)	3 (2.46%)	3 (2.46%)	3 (2.46%)	3 (2.46%)
*E. cloacae* (*n* = 3)	1 (0.82%)	2 (1.64%)	1 (0.82%)	2 (1.64%)	0 (0%)	2 (1.64%)	3 (2.46%)	3 (2.46%)	3 (2.46%)
**T**otal (*n* = 122)	23 (18.85%)	99 (81.15%)	14 (11.48%)	108 (88.52%)	23 (18.85%)	70 (57.38%)	114 (93.44%)	114 (93.44%)	106 (86.89%)

^*a*^ mCIM, modified carbapenem inactivation method; MHT, modified Hodge test; eCIM, EDTA-modified carbapenem inactivation method; n, number 254 of isolates; No., number; MBLs, metallo-β-lactamases.

### Analysis of the β5–β6 loop in OXA-48-like

Genomic bacteria amplified the β5–β6 loop, which comprises the catalytic part of the OXA-48-like enzyme against β-lactams (Fig. S1). PCR amplicons were subjected to nucleotide sequencing (Fig. S2).

**Fig 3:**
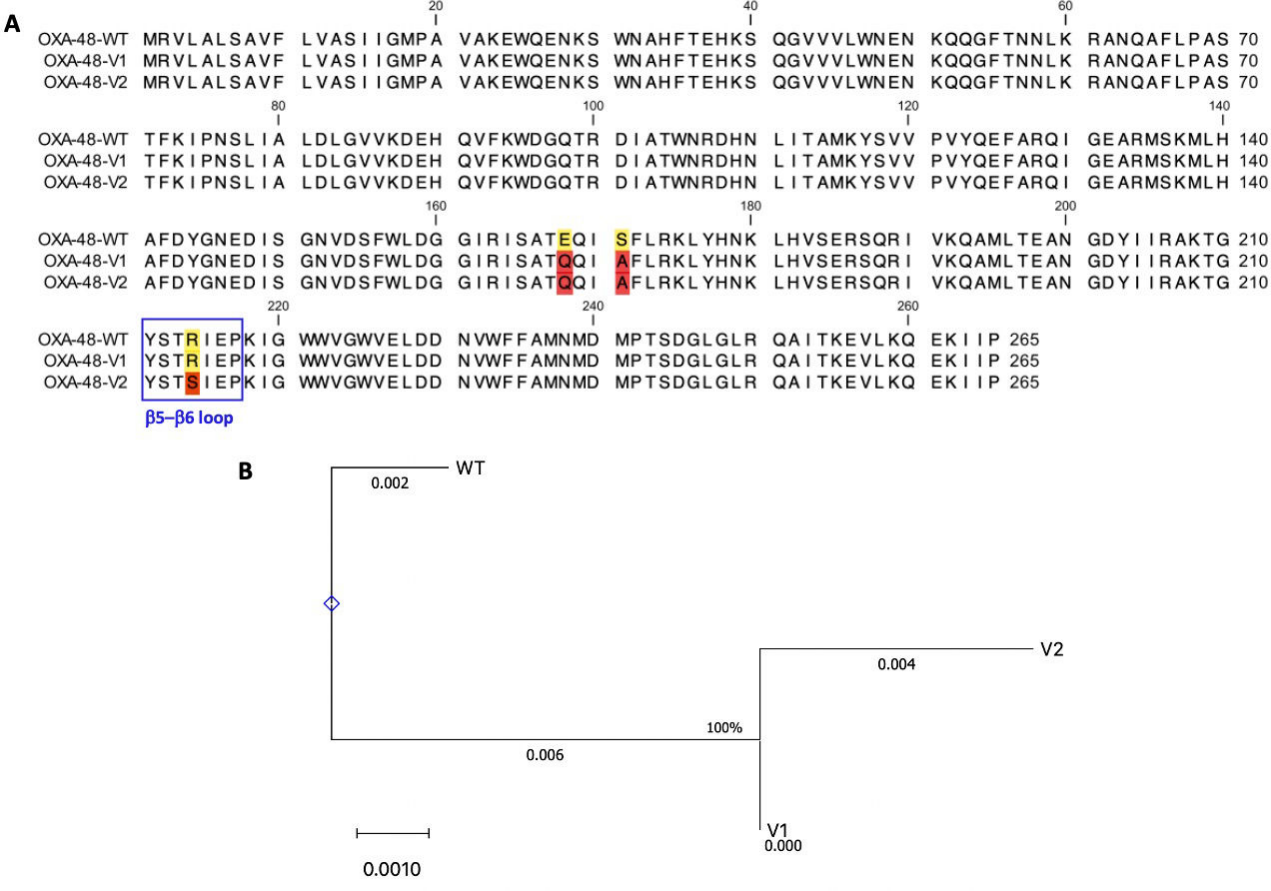
(**A**) Alignment of the amino acid sequence for OXA-48-like point mutations. The β5–β6 loop is framed in black for class D lactamases. Numbers were assigned using an OXA-48-like sequence. (**B**) Cladogram of the amino acid alignment of different OXA-48-like enzymes using the ClustalW2 sequence alignment program. Comparison of the deduced amino acid sequences among the OXA-48-like genes of the representative pattern of CRE isolates (wild type, V1, and V2).

Three-point mutations were identified in OXA48-like, including E168Q in 114 isolates (93.44%), S171A in 114 isolates (93.44%), and R214S in 106 isolates (86.89%). In this study, the enzyme mutations were classified into three types, namely WT [seven isolates (5.74%)], mutation 1 [V1 (E168Q/S171A), eight isolates (6.56%)], and mutation 2 [V2 (E168Q/S171A/R214S), 107 isolates (87.70%)]. The multiple sequence alignment and phylogenetic tree of the OXA-48-like mutation groups are depicted in Fig. 3A.

### Computerized simulation of mutations within the β5–β6 loop of OXA-48-like

Homology modeling of the OXA-48-like enzyme produced reliable Ramachandran plots. The percentages of WT, V1, and V2 residues in the most favored regions were 97.92%, 0.71%, and 0.00%, respectively. The percentages of WT, V1, and V2 residues in rotamer outliers were 98.13%, 0.71%, and 0.00%, respectively, whereas those in Ramachandran outlier regions were 98.34%, 0.71%, and 0.00%, respectively (Fig. S1B).

**Fig 4:**
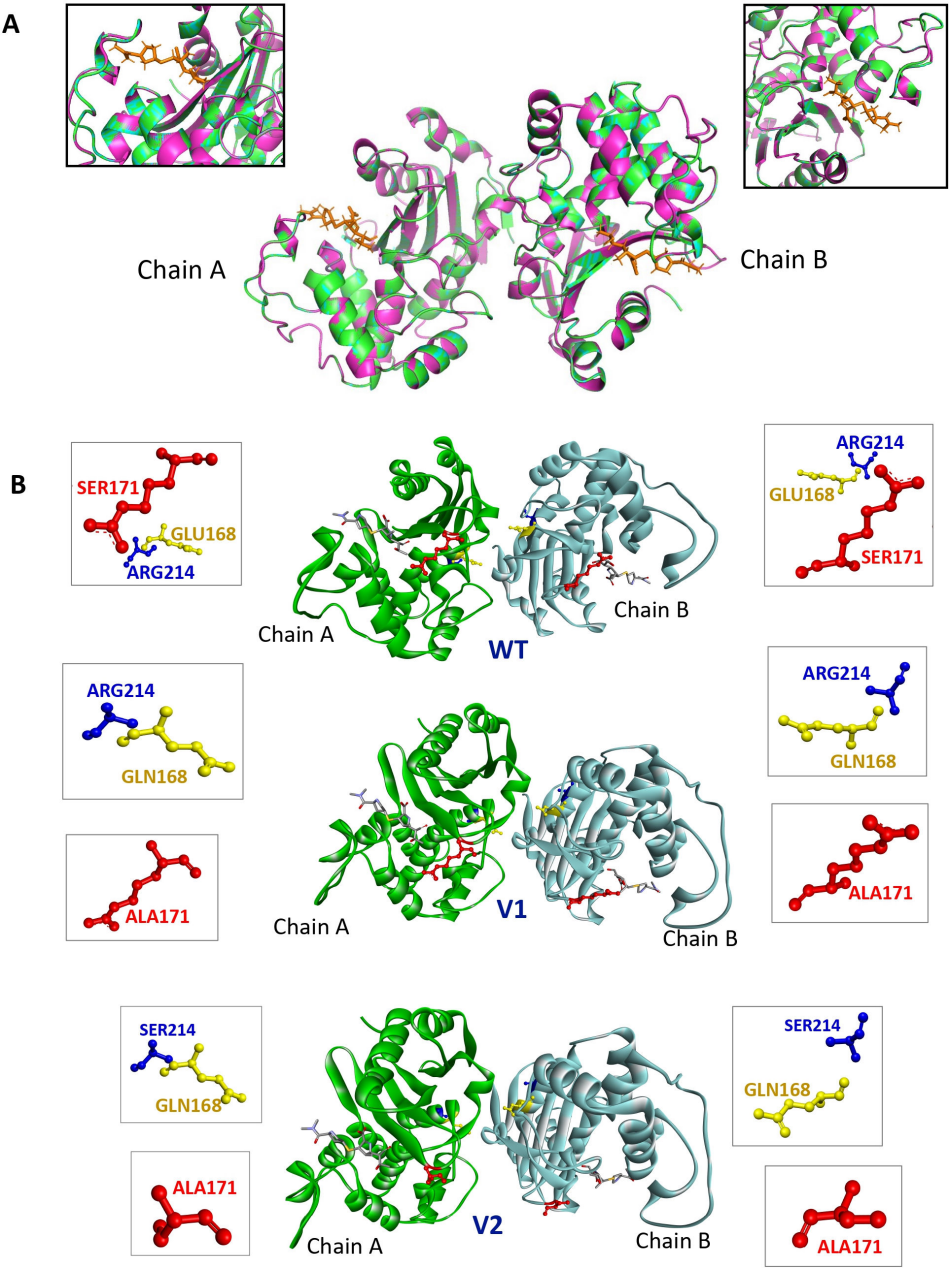
(**A**) Superposition of OXA-48-like WT (magenta), V1 (green), and V2 (cyan), which are magnified in the upper part of the figure. (**B**) Surface illustration of the OXA-48-WT, OXA-48- V1, and OXA-48-V2 models with the meropenem-binding site. Mutated amino acids (E168, yellow; S171, blue; R214, red) are shown in stick representation. The meropenem ligand is depicted in gray.

The *in silico* study was performed using pairwise structure superposition, which was performed using the CLICK server. The percent coverages of the overlapping structures between the modeled WT and V1, as well as the modeled WT and V2, indicated almost perfect superpositions of the key binding site residues of each pair (Table S1). Amino acid substitution in OXA-48-like located in β5–β6 might enhance the catalytic activity of the enzyme for meropenem. The results of superimposition and the surface illustration of the OXA-48-like mutation groups are presented in Fig. 4.

## DISCUSSION

Currently, as carbapenems represent the primary treatments for infections caused by multidrug-resistant pathogens, it is imperative to employ effective treatment options, especially for *Enterobacterales*. However, the inappropriate use of antibiotics has led to the emergence of carbapenem resistance through carbapenemase production, including class D OXA-48 in Gram-negative bacteria, which has become a significant concern in clinical practice. Monitoring of carbapenemases both in genotypic and phenotypic manner is often neglected due to the low MICs of carbapenems and also requires another strategy in addition to routine susceptibility testing (35, 36). In this study, we investigated *bla*_OXA-48-like_ variants/point mutations in CPE isolates and provided a new context for laboratory detection and therapeutic management. These variants or point mutations pose a significant risk to patients with infections attributed to these isolates (37). To the best of our knowledge, few official reports have described CPE isolates carrying variants or point mutations OXA-48-like in our country (38–40).

This study demonstrated a high prevalence of carbapenem resistance among *Enterobacterales* isolates, with *K. pneumoniae* and *E. coli* being the predominant species exhibiting carbapenem resistance. This finding aligns with previous global and regional reports identifying *K. pneumoniae* and *E. coli* as the major plasmid-mediated species harboring the carbapenem-resistant *bla*_OXA-48-like_ gene among CPE isolates, along with *Enterobacter* spp. (41, 42). A sizeable proportion of isolates (81.15%) were identified as metallo-β-lactamase (MBL) producers, including *E. coli* (29.51%), *K. pneumoniae* (23.77%), *E. aerogenes* (2.46%), and *E. cloacae* (1.64%). The high prevalence of MBL-producing *Enterobacterales* observed in our study is concerning and reflects the global challenge posed by these organisms, which are associated with limited treatment options and potential for rapid dissemination. The diverse *in vitro* sensitivity profiles observed for carbapenems and the classification of isolates as resistant or indeterminate to imipenem and meropenem can be attributed to the multidrug resistance conferred by the synthesis of extended-spectrum β-lactamases (ESBLs) and the presence of various carbapenemases. Accurate detection of carbapenemase production is crucial for guiding appropriate antimicrobial therapy and implementing effective infection control measures (43).

The MHT, recommended by CLSI for detecting CPE, is a simple and widely implemented phenotypic method in clinical laboratories. However, while it has a high sensitivity for detecting class A (KPC) and class D (OXA-48) carbapenemases, the MHT does not reliably identify MBL producers (44). The mCIM and eCIM employed in our study effectively identified 76.23% of isolates as carbapenemase producers, including both serine carbapenemases (18.85%) and the substantial proportion of MBL producers mentioned earlier. These phenotypic methods serve as valuable screening tools for carbapenemase production, complementing molecular techniques that provide definitive identification of specific carbapenemase variants/point mutations. The mCIM and eCIM serve as valuable screening tools for carbapenemase production, which is crucial for guiding appropriate antimicrobial therapy and implementing infection control measures (45–47).

Molecular studies enabled the definitive identification of OXA-48-like CPE in our setting. Given the current global emergence of this enzyme group, with OXA-48 and OXA-163 being the most prevalent, it is crucial to characterize the specific variants/point mutations circulating. The 11 OXA-48-like mutation groups identified to date exhibit varying hydrolytic activities against carbapenems, geographical distributions, associations with particular bacterial species, and resistance mechanisms (47).

In our study, we identified two distinct OXA-48-like point mutations harboring mutations found in OXA-181 (E168Q and S171A) and OXA-232 (R214S). The OXA-181 with E168Q and S171A mutations was previously reported in *K. pneumoniae* isolates from India. Similarly, the R214S mutation found in the OXA-232 was initially identified in a *K. pneumoniae* isolate recovered from a patient transferred from India to Mauritius in France (48).

The R214S substitution is located in the β5-β6 loop region, which is known to significantly influence the hydrolytic nature of class D β-lactamases (49). Modifications in this loop can alter the hydrolytic profile, potentially converting non-carbapenemases into carbapenemases, or vice versa (50). Such mutations may enable advantageous contacts with specific substrates or alleviate steric hindrance for substrate binding, leading to changes in the β-lactam spectrum of hydrolysis (51). The β5-β6 loop of OXA-48-like enzymes inhibits the binding of expanded-spectrum cephalosporins, while its effect on the rate of carbapenem turnover is variable—it may either decrease carbapenem turnover by hindering substrate binding or potentially increase carbapenem turnover by facilitating catalytic efficiency and promoting substrate interactions, depending on the specific structural and biochemical properties of the enzyme (49).

The identification of these OXA-48-like variants/point mutations in our setting, particularly their associations with *K. pneumoniae* isolates and potential links to the Indian subcontinent, highlights the need for continued molecular surveillance and epidemiological investigations. Importantly, mutations or deletions within the β5-β6 loop can potentially alter these effects. For instance, certain modifications in this region have been observed to enhance the enzyme ability to hydrolyze expanded-spectrum cephalosporins or increase the rate of carbapenem hydrolysis. Conversely, other changes may diminish or abolish the enzyme carbapenemase activity. Therefore, the β5-β6 loop acts as a critical structural determinant of OXA-48-like enzymes substrate specificity and catalytic efficiency, particularly regarding their interactions with expanded-spectrum cephalosporins and carbapenems.

This study reveals that out of 114 bacterial isolates identified with three-point mutations in OXA48-like genes, only 108 tested positive for carbapenemase production using the MHT. This situation highlights an interesting observation. While all isolates with three-point mutations in OXA48-like were expected to exhibit carbapenemase activity, not all of them tested positive in the MHT. Several factors could contribute to this discrepancy, such as the sensitivity of detection methods, the presence of non-functional mutations, and other mechanisms of carbapenem resistance. Overall, the finding that not all isolates with three-point mutations in OXA48-like tested positive in the MHT underscores the importance of using multiple detection methods and considering various factors that may influence test results when assessing carbapenemase production in clinical isolates (7, 8, 24).

Monitoring mutations in this region can provide insights into the potential impact on antimicrobial resistance profiles and guide appropriate therapeutic strategies. Clonal molecular research is necessary to further elucidate the origin, spread, and molecular epidemiology of OXA-48-like CPE infections in our region and globally.

Antibiotic stewardship is critical to prevent the emergence of bacterial resistance. The appropriate use of antimicrobial control systems in clinical settings reduces the need for carbapenems, thereby reducing the rate of carbapenem resistance. This approach has also displayed an association with a decline in the number of CRE isolates. Nevertheless, further analysis of the data is essential to ascertain the full impact of manipulating prescriptions for CRE strains (52). Addressing antimicrobial resistance necessitates adopting the “one health” approach and recognizing the interconnectedness of humans, animals, and the environment as potential sources of CPE infections. In this context, ongoing education and vigilant surveillance of CPE are imperative for preventing and controlling its transmission in healthcare facilities. This effort should engage all healthcare professionals in both direct and indirect contact with patients. In addition, it is advisable to implement programs focusing on research and epidemiological surveillance, enhancing the involvement of clinical laboratories through the development of flowcharts and the characterization of CPE. Surveillance culture tailored to local epidemiology should be used as a tool for informed decision-making based on the generated data (53). Our study had multiple limitations, indicating the need for a prospective evaluation with a larger sample size. The small number of cases is attributed to the novelty of emerging infections by CPE carrying *bla*_OXA-48-like_ variants/point mutations in our facility. Unfortunately, prospective analyses were not conducted because of constraints posed by our limited laboratory resources.

Our findings suggest a high prevalence of CPE based on phenotypic detection. In addition, we investigated OXA-48-like enzyme mutations. Clarification of the mechanisms responsible for carbapenem resistance in *Enterobacterales* has significant clinical implications, and research, including *in silico* studies, is needed to facilitate the development of preventive strategies and personalized antibiotic therapies. Considering the implications for infection control practices, antimicrobial stewardship, and public health interventions, ongoing surveillance is necessary to monitor changes in OXA-48-like CPE prevalence and characteristics over time.

## Data Availability

The data supporting the findings of this study, including the genomic sequences and homology modeling results, are publicly available. The blaOXA-48-like amplicon sequences have been deposited in the National Center for Biotechnology Information (NCBI) under accession number NG_049762.1. The structural data for the OXA-48-like wild-type enzyme complexed with meropenem is available in the Protein Data Bank (PDB) under accession ID 6ZRP. The predictive homology modeling and superimposition analyses were conducted using SWISS-MODEL (https://swissmodel.expasy.org) and PyMOL Molecular Graphics System, Version 2.0 (Schrodinger, LLC). The 3D structures generated in this study were evaluated using Ramachandran plots and further analyzed with the CLICK server (http://mspc.bii.a-star.edu.sg/click) for structural comparison. For any additional data requests or further inquiries, please contact the corresponding author.

## SUPPLEMENTAL MATERIAL

Supplemental material spectrum.00198-24-s0001.pdf Fig. S1 and S2; Table S1.

Supplemental data spectrum.00198-24-s0002.pdf Nucleotide sequence alignment.

ASM does not own the copyrights to Supplemental Material that may be linked to, or accessed through, an article. The authors have granted ASM a non-exclusive, world-wide license to publish the Supplemental Material files. Please contact the corresponding author directly for reuse.
