# Supplementary material for: Evaluation of blaOXA-48-like point mutation carbapenemase-producing Enterobacterales in Prapokklao Hospital, Thailand
Source: Microbiol Spectr. 2024 Oct 17;12(12):e00198-24. doi: 10.1128/spectrum.00198-24 (PMC11619526; doi:10.1128/spectrum.00198-24)
Supplement: Supplemental material — Fig. S1 and S2; Table S1. [file spectrum.00198-24-s0001.pdf]

Supplementary data

Emergence of *Bla*OXA-48-like point mutation Carbapenemase-producing *Enterobacterales* (CPE) recovered from Prapokklao Hospital, Thailand

Sirijan Santajit [1,2], Witawat Tunyongs[3], Thida Kong-Ngoen[3], Weewan Arsheewa[4], Woranich Hinthong[5,6] Pornpan Pumirat[3], Nitat Sookrung [7,8], and Nitaya Indrawattana [7,8,*]

[1] *Department of Medical Technology, School of Allied Health Sciences, Walailak University, Nakhon Si Thammarat 80160, Thailand*
[2] *Research Center in Tropical Pathobiology, Walailak University, Nakhon Si Thammarat 80160, Thailand*
[3] *Department of Microbiology and Immunology, Faculty of Tropical Medicine, Mahidol University, Bangkok 10400, Thailand*
[4] *Department of Microbiology, Prapokklao Hospital, Chanthaburi 22000, Thailand*
[5] *Princess Srisavangavadhana College of Medicine, Chulabhorn Royal Academy, Bangkok 10210, Thailand*
[6] *Department of Infection Biology, Faculty of Infectious and Tropical Diseases, London School of Hygiene and Tropical Medicine, WC1E 7HT, London, UK*
[7] *Siriraj Center of Research Excellence in Allergy and Immunology, Faculty of Medicine Siriraj Hospital, Mahidol University, Bangkok 10700, Thailand*
[8] *Biomedical Research Incubator Unit, Department of Research, Faculty of Medicine Siriraj Hospital, Mahidol University, Bangkok 10700, Thailand*

* Correspondence to: Biomedical Research Incubator Unit, Department of Research, Faculty of Medicine Siriraj Hospital, Mahidol University, Thailand.
E-mail address: nitaya.ind@mahidol.ac.th (N. Indrawattana).

WT

A
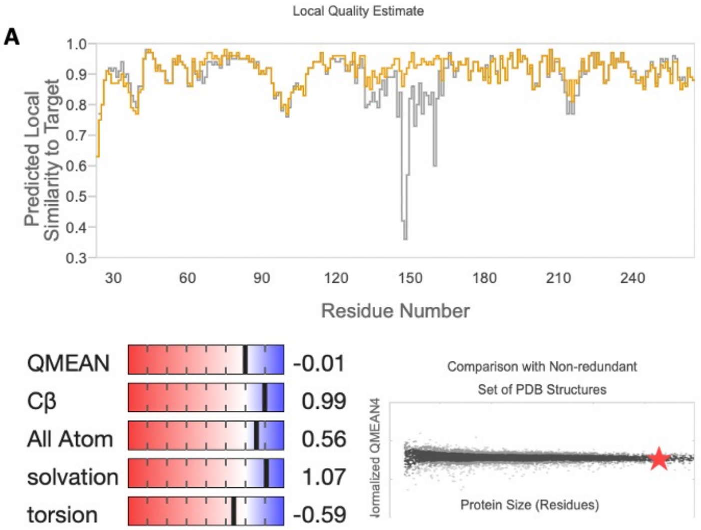

B
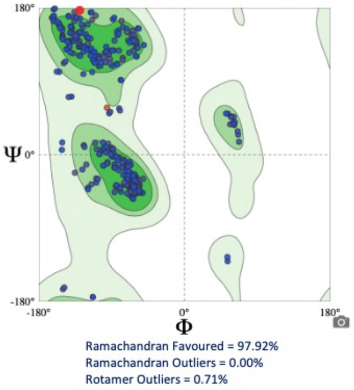

C
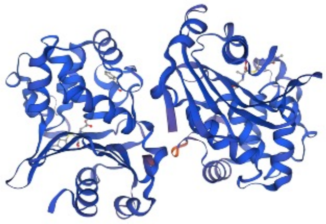

Template:6zrp.2.A Beta-lactamase
Crystal structure of class D Beta-lactamase OXA-48 in complex with meropenem
Seq Identity = 100.00%

V1

A
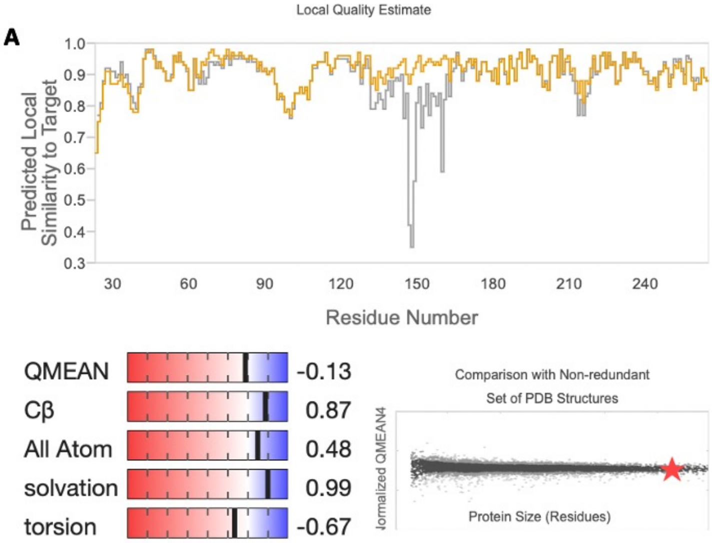

B
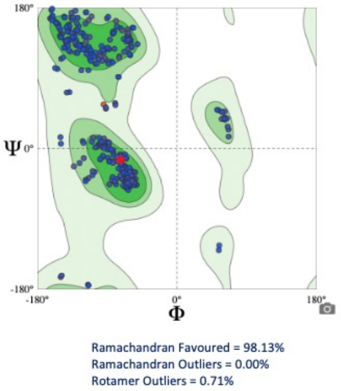

C
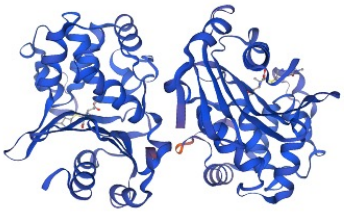

Template: 6zrp.2.A Beta-lactamase
Crystal structure of class D Beta-lactamase OXA-48 in complex with meropenem
Seq Identity = 99.25%

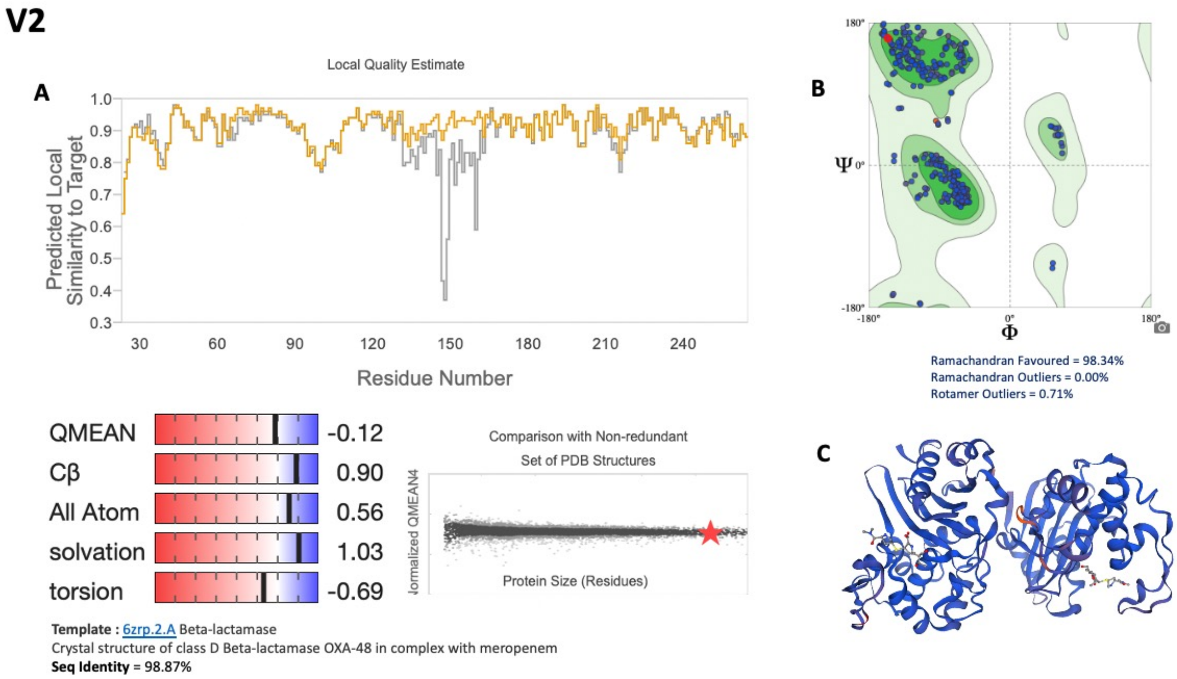

Supplementary Fig. S1 Validation of the homology modelling of OXA_48-like enzyme and its variants.

(A) Verify 3D graph plot for homology model validation (upper). Plot showing the QMEAN value as well as Z-score (lower left) and density plot for QMEAN showing the value of Z-score and QMEAN score (lower right). **(B)** Ramachandran plot statistics of the OXA-48-WT, OXA-48-V1 and OXA-48-V2. The most favored regions, additional allowed regions, generously allowed regions and disallowed regions are colored in red, yellow, light yellow and white, respectively. **(C)** Architecture of the OXA-48-WT, OXA-48-V1 and OXA-48-V2 models with the meropenem.

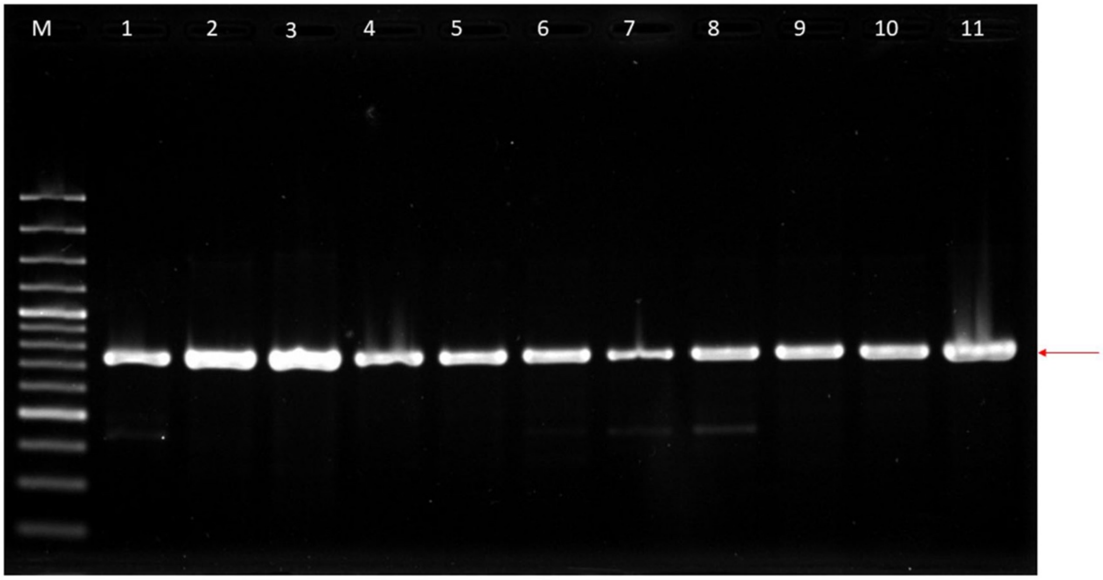

Supplementary Fig. S2 PCR amplicons of carbapenemase encoding genes.

Lane M, Marker ladder 100 base pairs plus. Lanes 1-11, the representatives of the positive CRE isolates of Carbapenemase encoding $bla_{OXA\text{-}48\text{-}like}$ was 743 bp. Numbers at the left and right are DNA masses in base pairs (bp)

Supplementary Table S1 The percentages of overlapping structures between the OXA-48-like wild type and its variants.

OXA-48-likeProteins	Percent overlapped structure	RMSD	Fragment score	Topology score	Number of identical residues	Z-score
WT-V1	100.00	0.02	1.00	1.00	481	21.17
WT-V2	100.00	0.02	1.00	1.00	479	21.17
