# Supplementary material for: Evaluation of blaOXA-48-like point mutation carbapenemase-producing Enterobacterales in Prapokklao Hospital, Thailand
Source: Microbiol Spectr. 2024 Oct 17;12(12):e00198-24. doi: 10.1128/spectrum.00198-24 (PMC11619526; doi:10.1128/spectrum.00198-24)
Supplement: Supplemental data — Nucleotide sequence alignment. [file spectrum.00198-24-s0002.pdf]

Supplementary data

Emergence of *Bla*OXA-48-like point mutation Carbapenemase-producing *Enterobacterales* (CPE) recovered from Prapokklao Hospital, Thailand

Sirijan Santajit [1,2], Witawat Tunyongs[3], Thida Kong-Ngoen[3], Weewan Arsheewa[4], Woranich Hinthong[5,6] Pornpan Pumirat[3], Nitat Sookrung [7,8], and Nitaya Indrawattana [7,8,*]

1 *Department of Medical Technology, School of Allied Health Sciences, Walailak University, Nakhon Si Thammarat 80160, Thailand*
2 *Research Center in Tropical Pathobiology, Walailak University, Nakhon Si Thammarat 80160, Thailand*
3 *Department of Microbiology and Immunology, Faculty of Tropical Medicine, Mahidol University, Bangkok 10400, Thailand*
4 *Department of Microbiology, Prapokklao Hospital, Chanthaburi 22000, Thailand*
5 *Princess Srisavangavadhana College of Medicine, Chulabhorn Royal Academy, Bangkok 10210, Thailand*
6 *Department of Infection Biology, Faculty of Infectious and Tropical Diseases, London School of Hygiene and Tropical Medicine, WC1E 7HT, London, UK*
7 *Siriraj Center of Research Excellence in Allergy and Immunology, Faculty of Medicine Siriraj Hospital, Mahidol University, Bangkok 10700, Thailand*
8 *Biomedical Research Incubator Unit, Department of Research, Faculty of Medicine Siriraj Hospital, Mahidol University, Bangkok 10700, Thailand*

* Correspondence to: Biomedical Research Incubator Unit, Department of Research, Faculty of Medicine Siriraj Hospital, Mahidol University, Thailand.
E-mail address: nitaya.ind@mahidol.ac.th (N. Indrawattana).

Consensus

1. 1st_BASE_4044639_F-63_F-OXA48
2. 1st_BASE_4044643_F-98_F-OXA48
3. 1st_BASE_4044644_F-100_F-OXA48
4. 1st_BASE_4044652_F-162_F-OXA48
5. 1st_BASE_4044680_F-40_F-OXA48
6. 1st_BASE_4044688_F-13_F-OXA48
7. 1st_BASE_4046666_F-154_B_F-OXA48
8. 1st_BASE_4044650_F-152_F-OXA48
9. 1st_BASE_4044672_F-120_F-OXA48
10. 1st_BASE_4044681_F-42_F-OXA48
11. 1st_BASE_4044684_F-154_A_F-OXA48
12. 1st_BASE_4044660_F-293_F-OXA48
13. 1st_BASE_4044646_F-111_F-OXA48
14. 1st_BASE_4044653_F-170_F-OXA48
15. 1st_BASE_4044654_F-184_F-OXA48
16. 1st_BASE_4044655_F-195_F-OXA48
17. 1st_BASE_4044664_F-334_F-OXA48
18. 1st_BASE_4044667_F-481_F-OXA48
19. 1st_BASE_4044641_F-91_F-OXA48
20. 1st_BASE_4044674_F-255_F-OXA48
21. 1st_BASE_4044657_F-230_F-OXA48
22. 1st_BASE_4044640_F-86_F-OXA48
23. 1st_BASE_4044671_F-77_F-OXA48
24. 1st_BASE_4044675_F-260_F-OXA48
25. 1st_BASE_4044673_F-233_F-OXA48
26. 1st_BASE_4044676_F-270_F-OXA48
27. 1st_BASE_4046665_F-137_B_F-OXA48
28. 1st_BASE_4044648_F-141_F-OXA48
29. 1st_BASE_4044662_F-307_F-OXA48
30. 1st_BASE_4044659_F-288_F-OXA48
31. 1st_BASE_4044679_F-38_F-OXA48
32. 1st_BASE_4044661_F-303_F-OXA48
33. 1st_BASE_4044663_F-327_F-OXA48
34. 1st_BASE_4044677_F-25_F-OXA48
35. 1st_BASE_4044685_F-275_F-OXA48
36. 1st_BASE_4044686_F-278_F-OXA48
37. 1st_BASE_4044647_F-137_A_F-OXA48
38. 1st_BASE_4044645_F-109_F-OXA48
39. 1st_BASE_4044656_F-212_F-OXA48
40. 1st_BASE_4044666_F-453_F-OXA48
41. 1st_BASE_4044665_F-442_F-OXA48
42. 1st_BASE_4044649_F-142_F-OXA48
43. 1st_BASE_4044670_F-73_F-OXA48
44. 1st_BASE_4044678_F-33_F-OXA48
45. 1st_BASE_4044682_F-96_F-OXA48
46. 1st_BASE_4044642_F-93_F-OXA48
47. 1st_BASE_4044689_F-15_F-OXA48
48. 1st_BASE_4044687_F-117_F-OXA48
49. 1st_BASE_4044637_F-7_F-OXA48
50. 1st_BASE_4044658_F-235_F-OXA48.ab1
51. 1st_BASE_4044638_F-10_F-OXA48
52. 1st_BASE_4044668_F-32_F-OXA48
53. 1st_BASE_4044669_F-72_F-OXA48

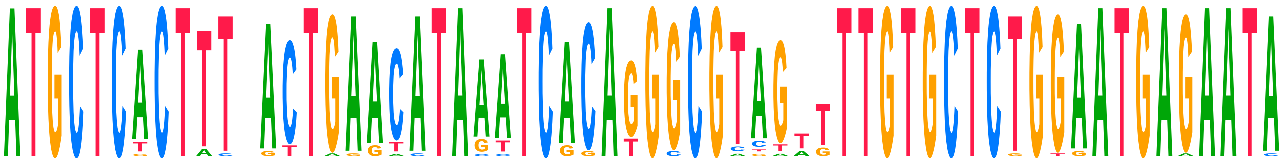

ATGCTCACTTT-ACTGAACATAAATCACAGGGCGTAG--TTGTGCTCTGGAATGAGAATA

1	ATGCTCACTTT-ACTGAACATAAATCACAGGGCGTAG--TTGTGCTCTGGAATGAGAATA	57
2	ATGCTCACTTT-ACTGAACATAAATCACAGGGCGTAG--TTGTGCTCTGGAATGAGAATA	57
3	ATGCTCACTTT-ACTGAACATAAATCACATGGCGTAG--TTGTGCTCTGGAATGAGAATA	57
4	ATGCTCACTTT-ACTGAACATAAATCACAGGGCGTAG--TTGTGCTCTGGAATGAGAATA	57
5	ATGCTCACTTT-ACTGAACATAAATCACAGGGCGTAG--TTGTGCTCTGGAATGAGAATA	57
6	ATGCTCACTTT-ACTGAACATAAATCACAGGGCGTAG--TTGTGCTCTGGAATGAGAATA	57
7	ATGCTCACTTT-ACTGAACATAAATCACAGGGCGTAG--TTGTGCTCTGGAATGAGAATA	57
8	ATGCTCACTTT-ACTGAACATAAATCACAGGGCGTAG--TTGTGCTCTGGAATGAGAATA	57
9	ATGCTCACTTT-ACTGAACATAAATCACAGGGCGTAG--TTGTGCTCTGGAATGAGAATA	57
10	ATGCTCACTTT-ACTGAACATAAATCACATGGCGTAG--TTGTGCTCTGGAATGAGAATA	57
11	ATGCTCACTTT-ACTGAACATAAATCACAGGGCGTAG--TTGTGCTCTGGAATGAGAATA	57
12	ATGCTCACTTT-ACTGAACATAAATCACAGGGCGTAG--TTGTGCTCTGGAATGAGAATA	57
13	ATGCTCACTTT-ACTGAACATAAATCACAGGGCGTAG--TTGTGCTCTGGAATGAGAATA	57
14	ATGCTCACTTT-ACTGAACATAAATCACAGGGCGTAG--TTGTGCTCTGGAATGAGAATA	57
15	ATGCTCACTTT-ACTGAACATAAATCACAGGGCGTAG--TTGTGCTCTGGAATGAGAATA	57
16	ATGCTCACTTT-ACTGAACATAAATCACAGGGCGTAG--TTGTGCTCTGGAATGAGAATA	57
17	ATGCTCACTTT-ACTGAACATAAATCACAGGGCGTAG--TTGTGCTCTGGAATGAGAATA	57
18	ATGCTCACTTT-ACTGAACATAAATCACAGGGCGTAG--TTGTGCTCTGGAATGAGAATA	57
19	ATGCTCACTTT-ACTGAACATAAATCACAGGGCGTAG--TTGTGCTCTGGAATGAGAATA	57
20	ATGCTCACTTT-ACTGAACATAAATCACAGGGCGTAG--TTGTGCTCTGGAATGAGAATA	57
21	ATGCTCACTTT-ACTGAACATAAATCACAGGGCGTAG--TTGTGCTCTGGAATGAGAATA	57
22	ATGCTCACTTT-ACTGAACATAAATCACAGGGCGTAG--TTGTGCTCTGGAATGAGAATA	57
23	ATGCTCACTTT-ACTGAACATAAATCACAGGGCGTAG--TTGTGCTCTGGAATGAGAATA	57
24	ATGCTCACTTT-ACTGAACATAAATCACAGGGCGTAG--TTGTGCTCTGGAATGAGAATA	57
25	ATGCTCACTTT-ACTGAACATAAATCACAGGGCGTAG--TTGTGCTCTGGAATGAGAATA	57
26	ATGCTCACTTT-ACTGAACATAAATCACAGGGCGTAG--TTGTGCTCTGGAATGAGAATA	57
27	ATGCTCACTTT-ACTGAACATANATCACAGGGCGTAG--TTGTGCTCTGGAATGAGAATA	57
28	ATGCTCACTTT-ACTGAACATAAATCACAGGGCGTAG--TTGTGCTCTGGAATGAGAATA	57
29	ATGCTCACTTT-ACTGAACATAAATCACAGGGCGTAG--TTGTGCTCTGGAATGAGAATA	57
30	ATGCTCACTTT-ACTGAACATAAATCACAGGGCGTAG--TTGTGCTCTGGAATGAGAATA	57
31	ATGCTCACTTT-ACTGAACATAAATCACATGGCGTAG--TTGTGCTCTGGAATGAGAATA	57
32	ATGCTCACTTT-ACTGAACATAAATCACAGGGCGTAG--TTGTGCTCTGGAATGAGAATA	57
33	ATGCTCACTTT-ACTGAACATAAATCACAGGGCGTAG--TTGTGCTCTGGAATGAGAATA	57
34	ATGCTCACTTT-ACTGAACATAAATCACAGGGCGTAG--TTGTGCTCTGGAATGAGAATA	57
35	ATGCTCACTTT-ACTGAACATAAATCACAGGGCGTAG--TTGTGCTCTGGAATGAGAATA	57
36	ATGCTCACTTT-ACTGAACATAAATCACAGGGCGTAG--TTGTGCTCTGGAATGAGAATA	57
37	ATGCTCACTTT-ACTGAACATAAATCACAGGGCGTAG--TTGTGCTCTGGAATGAGAATA	57
38	ATGCTCACTTT-ACTGAACATAAATCACAGGGCGTAG--TTGTGCTCTGGAATGAGAATA	57
39	ATGCTCACTTT-ACTGAACATAAATCACAGGGCGTAG--TTGTGCTCTGGAATGAGAATA	57
40	ATGCTCACTTT-ACTGAACATAAATCACAGGGCGTAG--TTGTGCTCTGGAATGAGAATA	57
41	ATGCTCACTTT-ACTGAACATAAATCACAGGGCGTAG--TTGTGCTCTGGAATGAGAATA	57
42	ATGCTCACTTT-ACTGAACATAGTTCACATGGCGCTT--TTGTGCTCTGGAATGAGAATA	57
43	ATGCTCACTTT-ACTGAACATAGATCGCATGGCGTAT--TTGTGCTCTGGAATGAGAATA	57
44	ATGCTCTCTTT-ATTGAGTATAGTTCGCATGGCGTN--TTTGTGCTCTGGAATGAGAATA	57
45	ATGCTCTCTAT-GTTGAGTATAGTTCGCATGCCGACATTTTGTGCTCTGGAATGAGAATA	59
46	-TGCTCTCTAT-GTTGAGTATAGTTCGCATGCCGTC-TTTTGTGCTCTGGAATGAGAATA	57
47	ATGCTCTCTTT-ATTGAGTATAGTTCGCATGGCGTC-TTTTGTGCTCTGGAATGAGAATA	58
48	ATGCTCTCTATTATTGAGTATAGTTCACATGGCGCA--TTTGTGCTCTGGAATGAGAATA	58
49	ATGCTCACTTT-ACTGAACATAAATCAGAGGGCGTAG--TTGTGCTCTGTGATGAGAATA	57
50	ATGCTCGCTTC-ACTAGAACTACCTCAGAGGG-GTGTAGTTGTGCTCGGGGATGAAAATC	58
51	ATGCTCACTTT-ACTGAACATAAATCACAGGGCGTAG--TTGTGCTCTGGAATGAGAATA	57
52	ATGCTCACTTT-ACTGAACATAAATCACAGGGCGTAG--TTGTGCTCTGGAATGAGAATA	57
53	ATGCTCACTTT-ACTGAACATAAATCACAGGGCGTAG--TTGTGCTCTGGAATGAGAATA	57

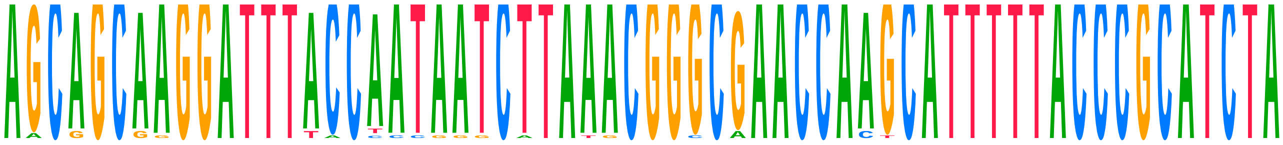

AGCAGCAAGGATTTACCAATAATCTTAAACGGGCGAACCAAGCATTTTTACCCGCATCTA

1	AGCAGCAAGGATTTACCAATAATCTTAAACGGGCGAACCAAGCATTTTTACCCGCATCTA	117
2	AGCAGCAAGGATTTACCAATAATCTTAAACGGGCGAACCAAGCATTTTTACCCGCATCTA	117
3	AGCAGCAAGGATTTACCAATAATCTTAAACGGGCGAACCAAGCATTTTTACCCGCATCTA	117
4	AGCAGCAAGGATTTACCAATAATCTTAAACGGGCGAACCAAGCATTTTTACCCGCATCTA	117
5	AGCAGCAAGGATTTACCAATAATCTTAAACGGGCGAACCAAGCATTTTTACCCGCATCTA	117
6	AGCAGCAAGGATTTACCAATAATCTTAAACGGGCGAACCAAGCATTTTTACCCGCATCTA	117
7	AGCAGCAAGGATTTACCAATAATCTTAAACGGGCGAACCAAGCATTTTTACCCGCATCTA	117
8	AGCAGCAAGGATTTACCAATAATCTTAAACGGGCGAACCAAGCATTTTTACCCGCATCTA	117
9	AGCAGCAAGGATTTACCAATAATCTTAAACGGGCGAACCAAGCATTTTTACCCGCATCTA	117
10	AGCAGCAAGGATTTACCAATAATCTTAAACGGGCGAACCAAGCATTTTTACCCGCATCTA	117
11	AGCAGCAAGGATTTACCAATAATCTTAAACGGGCGAACCAAGCATTTTTACCCGCATCTA	117
12	AGCAGCAAGGATTTACCAATAATCTTAAACGGGCGAACCAAGCATTTTTACCCGCATCTA	117
13	AGCAGCAAGGATTTACCAATAATCTTAAACGGGCGAACCAAGCATTTTTACCCGCATCTA	117
14	AGCAGCAAGGATTTACCAATAATCTTAAACGGGCGAACCAAGCATTTTTACCCGCATCTA	117
15	AGCAGCAAGGATTTACCAATAATCTTAAACGGGCGAACCAAGCATTTTTACCCGCATCTA	117
16	AGCAGCAAGGATTTACCAATAATCTTAAACGGGCGAACCAAGCATTTTTACCCGCATCTA	117
17	AGCAGCAAGGATTTACCAATAATCTTAAACGGGCGAACCAAGCATTTTTACCCGCATCTA	117
18	AGCAGCAAGGATTTACCAATAATCTTAAACGGGCGAACCAAGCATTTTTACCCGCATCTA	117
19	AGCAGCAAGGATTTACCAATAATCTTAAACGGGCGAACCAAGCATTTTTACCCGCATCTA	117
20	AGCAGCAAGGATTTACCAATAATCTTAAACGGGCGAACCAAGCATTTTTACCCGCATCTA	117
21	AGCAGCAAGGATTTACCAATAATCTTAAACGGGCGAACCAAGCATTTTTACCCGCATCTA	117
22	AGCAGCAAGGATTTACCAATAATCTTAAACGGGCGAACCAAGCATTTTTACCCGCATCTA	117
23	AGCAGCAAGGATTTACCAATAATCTTAAACGGGCGAACCAAGCATTTTTACCCGCATCTA	117
24	AGCAGCAAGGATTTACCAATAATCTTAAACGGGCGAACCAAGCATTTTTACCCGCATCTA	117
25	AGCAGCAAGGATTTACCAATAATCTTAAACGGGCGAACCAAGCATTTTTACCCGCATCTA	117
26	AGCAGCAAGGATTTACCAATAATCTTAAACGGGCGAACCAAGCATTTTTACCCGCATCTA	117
27	AGCAGCAAGGATTTACCAATAATCTTAAACGGGCGAACCAAGCATTTTTACCCGCATCTA	117
28	AGCAGCAAGGATTTACCAATAATCTTAAACGGGCGAACCAAGCATTTTTACCCGCATCTA	117
29	AGCAGCAAGGATTTACCAATAATCTTAAACGGGCGAACCAAGCATTTTTACCCGCATCTA	117
30	AGCAGCAAGGATTTACCAATAATCTTAAACGGGCGAACCAAGCATTTTTACCCGCATCTA	117
31	AGCAGCAAGGATTTACCAATAATCTTAAACGGGCGAACCAAGCATTTTTACCCGCATCTA	117
32	AGCAGCAAGGATTTACCAATAATCTTAAACGGGCGAACCAAGCATTTTTACCCGCATCTA	117
33	AGCAGCAAGGATTTACCAATAATCTTAAACGGGCGAACCAAGCATTTTTACCCGCATCTA	117
34	AGCAGCAAGGATTTACCAATAATCTTAAACGGGCGAACCAAGCATTTTTACCCGCATCTA	117
35	AGCAGCAAGGATTTACCAATAATCTTAAACGGGCGAACCAAGCATTTTTACCCGCATCTA	117
36	AGCAGCAAGGATTTACCAATAATCTTAAACGGGCGAACCAAGCATTTTTACCCGCATCTA	117
37	AGCAGCAAGGATTTACCAATAATCTTAAACGGGCGAACCAAGCATTTTTACCCGCATCTA	117
38	AGCAGCAAGGATTTACCAATAATCTTAAACGGGCGAACCAAGCATTTTTACCCGCATCTA	117
39	AGCAGCAAGGATTTACCAATAATCTTAAACGGGCGAACCAAGCATTTTTACCCGCATCTA	117
40	AGCAGCAAGGATTTACCAATAATCTTAAACGGGCGAACCAAGCATTTTTACCCGCATCTA	117
41	AGCAGCAAGGATTTACCAATAATCTTAAACGGGCGAACCAAGCATTTTTACCCGCATCTA	117
42	AGCAGCAAGGATTTACCCATAATCTTAAACGGCCAAACCAAGCATTTTTACCCGCATCTA	117
43	AGCAGCAGGGATTTTCCAATAATCTTATGCGGGCGAACCAAGCATTTTTACCCGCATCTA	117
44	AGCAGCAAGGATTTTCCAATAATCTTAAACGGGCGAACCAAGCATTTTTACCCGCATCTA	117
45	AGCAGCAAGGATTTACCAATAATCTTAAACGGGCGAACCAAGCATTTTTACCCGCATCTA	119
46	AGCAGCAAGGATTTACCAATAATCTTAAACGGGCGAACCAAGCATTTTTACCCGCATCTA	117
47	AGCAGCAAGGATTTTCCAATAATCTTAAACGGGCGAACCAAGCATTTTTACCCGCATCTA	118
48	AGCAGCAAGGATTTACCAATAATCTTAAACGGGCGAACCAAGCATTTTTACCCGCATCTA	118
49	AACGGCGAGGATTTACCTCTAATCTTAAACGGGCGAACCACGCATTTTTACCCGCATCTA	117
50	AACGGCGAGGATTTAACT-CGGGCATAAACGGGCAAACCACTCATTTTTACCCGCATCTA	117
51	AGCAGCAAGGATTTACCAATAATCTTAAACGGGCGAACCAAGCATTTTTACCCGCATCTA	117
52	AGCAGCAAGGATTTACCAATAATCTTAAACGGGCGAACCAAGCATTTTTACCCGCATCTA	117
53	AGCGGCGAGGATTTACCAATAATCTTAAACGGGCAAACCACGCATTTTTACCCGCATCTA	117

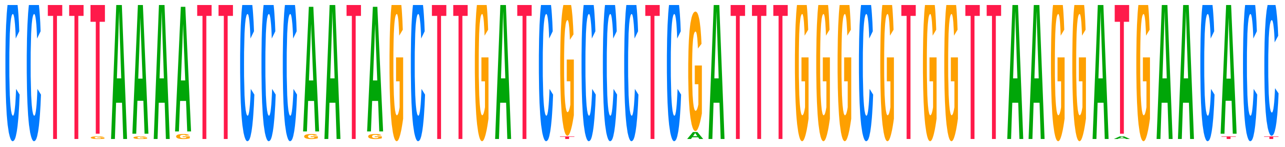

	Sequence	
	CCTTTAAAATTCCCAATAGCTTGATCGCCCTCGATTTGGGCGTGGTTAAGGATGAACACC	
1	CCTTTAAAATTCCCAATAGCTTGATCGCCCTCGATTTGGGCGTGGTTAAGGATGAACACC	177
2	CCTTTAAAATTCCCAATAGCTTGATCGCCCTCGATTTGGGCGTGGTTAAGGATGAACACC	177
3	CCTTTAAAATTCCCAATAGCTTGATCGCCCTCGATTTGGGCGTGGTTAAGGATGAACACC	177
4	CCTTTAAAATTCCCAATAGCTTGATCGCCCTCGATTTGGGCGTGGTTAAGGATGAACACC	177
5	CCTTTAAAATTCCCAATAGCTTGATCGCCCTCGATTTGGGCGTGGTTAAGGATGAACACC	177
6	CCTTTAAAATTCCCAATAGCTTGATCGCCCTCGATTTGGGCGTGGTTAAGGATGAACACC	177
7	CCTTTAAAATTCCCAATAGCTTGATCGCCCTCGATTTGGGCGTGGTTAAGGATGAACACC	177
8	CCTTTAAAATTCCCAATAGCTTGATCGCCCTCGATTTGGGCGTGGTTAAGGATGAACACC	177
9	CCTTTAAAATTCCCAATAGCTTGATCGCCCTCGATTTGGGCGTGGTTAAGGATGAACACC	177
10	CCTTTAAAATTCCCAATAGCTTGATCGCCCTCGATTTGGGCGTGGTTAAGGATGAACACC	177
11	CCTTTAAAATTCCCAATAGCTTGATCGCCCTCGATTTGGGCGTGGTTAAGGATGAACACC	177
12	CCTTTAAAATTCCCAATAGCTTGATCGCCCTCGATTTGGGCGTGGTTAAGGATGAACACC	177
13	CCTTTAAAATTCCCAATAGCTTGATCGCCCTCGATTTGGGCGTGGTTAAGGATGAACACC	177
14	CCTTTAAAATTCCCAATAGCTTGATCGCCCTCGATTTGGGCGTGGTTAAGGATGAACACC	177
15	CCTTTAAAATTCCCAATAGCTTGATCGCCCTCGATTTGGGCGTGGTTAAGGATGAACACC	177
16	CCTTTAAAATTCCCAATAGCTTGATCGCCCTCGATTTGGGCGTGGTTAAGGATGAACACC	177
17	CCTTTAAAATTCCCAATAGCTTGATCGCCCTCGATTTGGGCGTGGTTAAGGATGAACACC	177
18	CCTTTAAAATTCCCAATAGCTTGATCGCCCTCGATTTGGGCGTGGTTAAGGATGAACACC	177
19	CCTTTAAAATTCCCAATAGCTTGATCGCCCTCGATTTGGGCGTGGTTAAGGATGAACACC	177
20	CCTTTAAAATTCCCAATAGCTTGATCGCCCTCGATTTGGGCGTGGTTAAGGATGAACACC	177
21	CCTTTAAAATTCCCAATAGCTTGATCGCCCTCGATTTGGGCGTGGTTAAGGATGAACACC	177
22	CCTTTAAAATTCCCAATAGCTTGATCGCCCTCGATTTGGGCGTGGTTAAGGATGAACACC	177
23	CCTTTAAAATTCCCAATAGCTTGATCGCCCTCGATTTGGGCGTGGTTAAGGATGAACACC	177
24	CCTTTAAAATTCCCAATAGCTTGATCGCCCTCGATTTGGGCGTGGTTAAGGATGAACACC	177
25	CCTTTAAAATTCCCAATAGCTTGATCGCCCTCGATTTGGGCGTGGTTAAGGATGAACACC	177
26	CCTTTAAAATTCCCAATAGCTTGATCGCCCTCGATTTGGGCGTGGTTAAGGATGAACACC	177
27	CCTTTAAAATTCCCAATAGCTTGATCGCCCTCGATTTGGGCGTGGTTAAGGATGAACACC	177
28	CCTTTAAAATTCCCAATAGCTTGATCGCCCTCGATTTGGGCGTGGTTAAGGATGAACACC	177
29	CCTTTAAAATTCCCAATAGCTTGATCGCCCTCGATTTGGGCGTGGTTAAGGATGAACACC	177
30	CCTTTAAAATTCCCAATAGCTTGATCGCCCTCGATTTGGGCGTGGTTAAGGATGAACACC	177
31	CCTTTAAAATTCCCAATAGCTTGATCGCCCTCGATTTGGGCGTGGTTAAGGATGAACACC	177
32	CCTTTAAAATTCCCAATAGCTTGATCGCCCTCGATTTGGGCGTGGTTAAGGATGAACACC	177
33	CCTTTAAAATTCCCAATAGCTTGATCGCCCTCGATTTGGGCGTGGTTAAGGATGAACACC	177
34	CCTTTAAAATTCCCAATAGCTTGATCGCCCTCGATTTGGGCGTGGTTAAGGATGAACACC	177
35	CCTTTAAAATTCCCAATAGCTTGATCGCCCTCGATTTGGGCGTGGTTAAGGATGAACACC	177
36	CCTTTAAAATTCCCAATAGCTTGATCGCCCTCGATTTGGGCGTGGTTAAGGATGAACACC	177
37	CCTTTAAAATTCCCAATAGCTTGATCGCCCTCGATTTGGGCGTGGTTAAGGATGAACACC	177
38	CCTTTAAAATTCCCAATAGCTTGATCGCCCTCGATTTGGGCGTGGTTAAGGATGAACACC	177
39	CCTTTAAAATTCCCAATAGCTTGATCGCCCTCGATTTGGGCGTGGTTAAGGATGAACACC	177
40	CCTTTAAAATTCCCAATAGCTTGATCGCCCTCGATTTGGGCGTGGTTAAGGATGAACACC	177
41	CCTTTAAAATTCCCAATAGCTTGATCGCCCTCGATTTGGGCGTGGTTAAGGATGAACACC	177
42	CCTTTAAAATTCCCAATAGCTTGATCGCCCTCGATTTGGGCGTGGTTAAGGATGAACACC	177
43	CCTTTAAAATTCCCAATAGCTTGATCGCCCTCGATTTGGGCGTGGTTAAGGATGAACACC	177
44	CCTTTAAAATTCCCAATAGCTTGATCGCCCTCGATTTGGGCGTGGTTAAGGATGAACACC	177
45	CCTTTAAAATTCCCAATAGCTTGATCGCCCTCGATTTGGGCGTGGTTAAGGATGAACACC	179
46	CCTTTAAAATTCCCAATAGCTTGATCGCCCTCGATTTGGGCGTGGTTAAGGATGAACACC	177
47	CCTTTAAAATTCCCAATAGCTTGATCGCCCTCGATTTGGGCGTGGTTAAGGATGAACACC	178
48	CCTTTAAAATTCCCAATAGCTTGATCGCCCTCGATTTGGGCGTGGTTAAGGATGAACACC	178
49	CCTTGAGAGTTCCCGATGGCTTGATCGCCCTCAATTTGGGCGTGGTTAAGGATGAACACC	177
50	CCTTTAAAGTTCCCGATGGCTTGATCTCCCTCAATTTGGGCGTGGTTAAGGAAGAACACC	177
51	CCTTTAAAATTCCCAATAGCTTGATCGCCCTCGATTTGGGCGTGGTTAAGGATGAACACC	177
52	CCTTTAAAATTCCCAATAGCTTGATCGCCCTCGATTTGGGCGTGGTTAAGGATGAACACC	177
53	CCTTTAAAATTCCCAATAGCTTGATCGCCCTCAATTTGGGCGTGGTTAAGGATGAACTCT	177

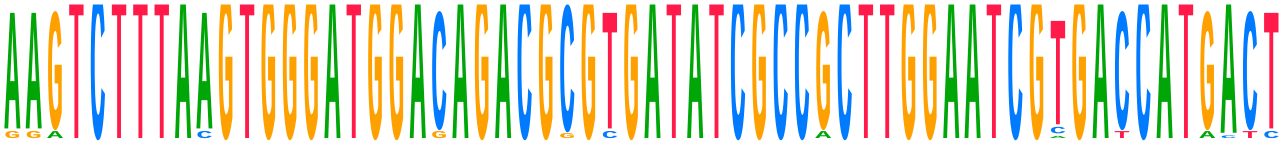

	AAGTCTTTAAGTGGGATGGACAGACGCGTGATATCGCCGCTTGGAATCGTGACCATGACT	
1	AAGTCTTTAAGTGGGATGGACAGACGCGTGATATCGCCGCTTGGAATCGTGACCATGACT	237
2	AAGTCTTTAAGTGGGATGGACAGACGCGTGATATCGCCGCTTGGAATCGTGACCATGACT	237
3	AAGTCTTTAAGTGGGATGGACAGACGCGTGATATCGCCGCTTGGAATCGTGACCATGACT	237
4	AAGTCTTTAAGTGGGATGGACAGACGCGTGATATCGCCGCTTGGAATCGTGACCATGACT	237
5	AAGTCTTTAAGTGGGATGGACAGACGCGTGATATCGCCGCTTGGAATCGTGACCATGACT	237
6	AAGTCTTTAAGTGGGATGGACAGACGCGTGATATCGCCGCTTGGAATCGTGACCATGACT	237
7	AAGTCTTTAAGTGGGATGGACAGACGCGTGATATCGCCGCTTGGAATCGTGACCATGACT	237
8	AAGTCTTTAAGTGGGATGGACAGACGCGTGATATCGCCGCTTGGAATCGTGACCATGACT	237
9	AAGTCTTTAAGTGGGATGGACAGACGCGTGATATCGCCGCTTGGAATCGTGACCATGACT	237
10	AAGTCTTTAAGTGGGATGGACAGACGCGTGATATCGCCGCTTGGAATCGTGACCATGACT	237
11	AAGTCTTTAAGTGGGATGGACAGACGCGTGATATCGCCGCTTGGAATCGTGACCATGACT	237
12	AAGTCTTTAAGTGGGATGGACAGACGCGTGATATCGCCGCTTGGAATCGTGACCATGACT	237
13	AAGTCTTTAAGTGGGATGGACAGACGCGTGATATCGCCGCTTGGAATCGTGACCATGACT	237
14	AAGTCTTTAAGTGGGATGGACAGACGCGTGATATCGCCGCTTGGAATCGTGACCATGACT	237
15	AAGTCTTTAAGTGGGATGGACAGACGCGTGATATCGCCGCTTGGAATCGTGACCATGACT	237
16	AAGTCTTTAAGTGGGATGGACAGACGCGTGATATCGCCGCTTGGAATCGTGACCATGACT	237
17	AAGTCTTTAAGTGGGATGGACAGACGCGTGATATCGCCGCTTGGAATCGTGACCATGACT	237
18	AAGTCTTTAAGTGGGATGGACAGACGCGTGATATCGCCGCTTGGAATCGTGACCATGACT	237
19	AAGTCTTTAAGTGGGATGGACAGACGCGTGATATCGCCGCTTGGAATCGTGACCATGACT	237
20	AAGTCTTTAAGTGGGATGGACAGACGCGTGATATCGCCGCTTGGAATCGTGACCATGACT	237
21	AAGTCTTTAAGTGGGATGGACAGACGCGTGATATCGCCGCTTGGAATCGTGACCATGACT	237
22	AAGTCTTTAAGTGGGATGGACAGACGCGTGATATCGCCGCTTGGAATCGTGACCATGACT	237
23	AAGTCTTTAAGTGGGATGGACAGACGCGTGATATCGCCGCTTGGAATCGTGACCATGACT	237
24	AAGTCTTTAAGTGGGATGGACAGACGCGTGATATCGCCGCTTGGAATCGTGACCATGACT	237
25	AAGTCTTTAAGTGGGATGGACAGACGCGTGATATCGCCGCTTGGAATCGTGACCATGACT	237
26	AAGTCTTTAAGTGGGATGGACAGACGCGTGATATCGCCGCTTGGAATCGTGACCATGACT	237
27	AAGTCTTTAAGTGGGATGGACAGACGCGTGATATCGCCGCTTGGAATCGTGACCATGACT	237
28	AAGTCTTTAAGTGGGATGGACAGACGCGTGATATCGCCGCTTGGAATCGTGACCATGACT	237
29	AAGTCTTTAAGTGGGATGGACAGACGCGTGATATCGCCGCTTGGAATCGTGACCATGACT	237
30	AAGTCTTTAAGTGGGATGGACAGACGCGTGATATCGCCGCTTGGAATCGTGACCATGACT	237
31	AAGTCTTTAAGTGGGATGGACAGACGCGTGATATCGCCGCTTGGAATCGTGACCATGACT	237
32	AAGTCTTTAAGTGGGATGGACAGACGCGTGATATCGCCGCTTGGAATCGTGACCATGACT	237
33	AAGTCTTTAAGTGGGATGGACAGACGCGTGATATCGCCGCTTGGAATCGTGACCATGACT	237
34	AAGTCTTTAAGTGGGATGGACAGACGCGTGATATCGCCGCTTGGAATCGTGACCATGACT	237
35	AAGTCTTTAAGTGGGATGGACAGACGCGTGATATCGCCGCTTGGAATCGTGACCATGACT	237
36	AAGTCTTTAAGTGGGATGGACAGACGCGTGATATCGCCGCTTGGAATCGTGACCATGACT	237
37	AAGTCTTTAAGTGGGATGGACAGACGCGTGATATCGCCGCTTGGAATCGTGACCATGACT	237
38	AAGTCTTTAAGTGGGATGGACAGACGCGTGATATCGCCGCTTGGAATCGTGACCATGACT	237
39	AAGTCTTTAAGTGGGATGGACAGACGCGTGATATCGCCGCTTGGAATCGTGACCATGACT	237
40	AAGTCTTTAAGTGGGATGGACAGACGCGTGATATCGCCGCTTGGAATCGTGACCATGACT	237
41	AAGTCTTTAAGTGGGATGGACAGACGCGTGATATCGCCGCTTGGAATCGTGACCATGACT	237
42	AAGTCTTTAAGTGGGATGGACAGACGCGTGATATCGCCGCTTGGAATCGTGACCATGACT	237
43	AAGTCTTTAAGTGGGATGGACAGACGCGTGATATCGCCGCTTGGAATCGTGACCATGACT	237
44	AAGTCTTTAAGTGGGATGGACAGACGCGTGATATCGCCGCTTGGAATCGTGACCATGACT	237
45	AAGTCTTTAAGTGGGATGGACAGACGCGTGATATCGCCGCTTGGAATCGTGACCATGACT	239
46	AAGTCTTTAAGTGGGATGGACAGACGCGTGATATCGCCGCTTGGAATCGTGACCATGACT	237
47	AAGTCTTTAAGTGGGATGGACAGACGCGTGATATCGCCGCTTGGAATCGTGACCATGACT	238
48	AAGTCTTTAAGTGGGATGGACAGACGCGTGATATCGCCGCTTGGAATCGTGACCATGACT	238
49	GGATCTTTACGTGGGATGGAGAGACGGGTGATATCGCCGCTTGGAATCGAGACCATGACT	237
50	GGATCTTTACGTGGGATGGAGAGACGGGTGATATCGCCGCTTGGAATCGTGACCATGCCT	237
51	AAGTCTTTAAGTGGGATGGACAGACGCGCGATATCGCCACTTGGAATCGCGATCATAATC	237
52	AAGTCTTTAAGTGGGATGGACAGACGCGCGATATCGCCACTTGGAATCGCGATCATAATC	237
53	GGATCTTTACGTGGGATGGAGAGACGCGCGATATCGCCACTTGGAATCGCGATCATAATC	237

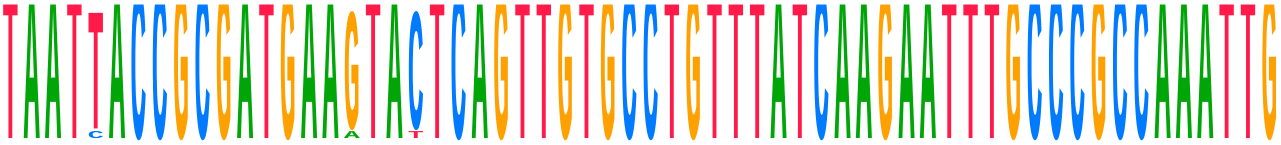

	TAATTACCGCGATGAAGTACTCAGTTGTGCCTGTTTATCAAGAATTTGCCCGCCAAATTG	
1	TAATTACCGCGATGAAGTACTCAGTTGTGCCTGTTTATCAAGAATTTGCCCGCCAAATTG	297
2	TAATTACCGCGATGAAGTACTCAGTTGTGCCTGTTTATCAAGAATTTGCCCGCCAAATTG	297
3	TAATTACCGCGATGAAGTACTCAGTTGTGCCTGTTTATCAAGAATTTGCCCGCCAAATTG	297
4	TAATTACCGCGATGAAGTACTCAGTTGTGCCTGTTTATCAAGAATTTGCCCGCCAAATTG	297
5	TAATTACCGCGATGAAGTACTCAGTTGTGCCTGTTTATCAAGAATTTGCCCGCCAAATTG	297
6	TAATTACCGCGATGAAGTACTCAGTTGTGCCTGTTTATCAAGAATTTGCCCGCCAAATTG	297
7	TAATTACCGCGATGAAGTACTCAGTTGTGCCTGTTTATCAAGAATTTGCCCGCCAAATTG	297
8	TAATTACCGCGATGAAGTACTCAGTTGTGCCTGTTTATCAAGAATTTGCCCGCCAAATTG	297
9	TAATTACCGCGATGAAGTACTCAGTTGTGCCTGTTTATCAAGAATTTGCCCGCCAAATTG	297
10	TAATTACCGCGATGAAGTACTCAGTTGTGCCTGTTTATCAAGAATTTGCCCGCCAAATTG	297
11	TAATTACCGCGATGAAGTACTCAGTTGTGCCTGTTTATCAAGAATTTGCCCGCCAAATTG	297
12	TAATTACCGCGATGAAGTACTCAGTTGTGCCTGTTTATCAAGAATTTGCCCGCCAAATTG	297
13	TAATTACCGCGATGAAGTACTCAGTTGTGCCTGTTTATCAAGAATTTGCCCGCCAAATTG	297
14	TAATTACCGCGATGAAGTACTCAGTTGTGCCTGTTTATCAAGAATTTGCCCGCCAAATTG	297
15	TAATTACCGCGATGAAGTACTCAGTTGTGCCTGTTTATCAAGAATTTGCCCGCCAAATTG	297
16	TAATTACCGCGATGAAGTACTCAGTTGTGCCTGTTTATCAAGAATTTGCCCGCCAAATTG	297
17	TAATTACCGCGATGAAGTACTCAGTTGTGCCTGTTTATCAAGAATTTGCCCGCCAAATTG	297
18	TAATTACCGCGATGAAGTACTCAGTTGTGCCTGTTTATCAAGAATTTGCCCGCCAAATTG	297
19	TAATTACCGCGATGAAGTACTCAGTTGTGCCTGTTTATCAAGAATTTGCCCGCCAAATTG	297
20	TAATTACCGCGATGAAGTACTCAGTTGTGCCTGTTTATCAAGAATTTGCCCGCCAAATTG	297
21	TAATTACCGCGATGAAGTACTCAGTTGTGCCTGTTTATCAAGAATTTGCCCGCCAAATTG	297
22	TAATTACCGCGATGAAGTACTCAGTTGTGCCTGTTTATCAAGAATTTGCCCGCCAAATTG	297
23	TAATTACCGCGATGAAGTACTCAGTTGTGCCTGTTTATCAAGAATTTGCCCGCCAAATTG	297
24	TAATTACCGCGATGAAGTACTCAGTTGTGCCTGTTTATCAAGAATTTGCCCGCCAAATTG	297
25	TAATTACCGCGATGAAGTACTCAGTTGTGCCTGTTTATCAAGAATTTGCCCGCCAAATTG	297
26	TAATTACCGCGATGAAGTACTCAGTTGTGCCTGTTTATCAAGAATTTGCCCGCCAAATTG	297
27	TAATTACCGCGATGAAGTACTCAGTTGTGCCTGTTTATCAAGAATTTGCCCGCCAAATTG	297
28	TAATTACCGCGATGAAGTACTCAGTTGTGCCTGTTTATCAAGAATTTGCCCGCCAAATTG	297
29	TAATTACCGCGATGAAGTACTCAGTTGTGCCTGTTTATCAAGAATTTGCCCGCCAAATTG	297
30	TAATTACCGCGATGAAGTACTCAGTTGTGCCTGTTTATCAAGAATTTGCCCGCCAAATTG	297
31	TAATTACCGCGATGAAGTACTCAGTTGTGCCTGTTTATCAAGAATTTGCCCGCCAAATTG	297
32	TAATTACCGCGATGAAGTACTCAGTTGTGCCTGTTTATCAAGAATTTGCCCGCCAAATTG	297
33	TAATTACCGCGATGAAGTACTCAGTTGTGCCTGTTTATCAAGAATTTGCCCGCCAAATTG	297
34	TAATTACCGCGATGAAGTACTCAGTTGTGCCTGTTTATCAAGAATTTGCCCGCCAAATTG	297
35	TAATTACCGCGATGAAGTACTCAGTTGTGCCTGTTTATCAAGAATTTGCCCGCCAAATTG	297
36	TAATTACCGCGATGAAGTACTCAGTTGTGCCTGTTTATCAAGAATTTGCCCGCCAAATTG	297
37	TAATTACCGCGATGAAGTACTCAGTTGTGCCTGTTTATCAAGAATTTGCCCGCCAAATTG	297
38	TAATTACCGCGATGAAGTACTCAGTTGTGCCTGTTTATCAAGAATTTGCCCGCCAAATTG	297
39	TAATTACCGCGATGAAGTACTCAGTTGTGCCTGTTTATCAAGAATTTGCCCGCCAAATTG	297
40	TAATTACCGCGATGAAGTACTCAGTTGTGCCTGTTTATCAAGAATTTGCCCGCCAAATTG	297
41	TAATTACCGCGATGAAGTACTCAGTTGTGCCTGTTTATCAAGAATTTGCCCGCCAAATTG	297
42	TAATTACCGCGATGAAGTACTCAGTTGTGCCTGTTTATCAAGAATTTGCCCGCCAAATTG	297
43	TAATTACCGCGATGAAGTACTCAGTTGTGCCTGTTTATCAAGAATTTGCCCGCCAAATTG	297
44	TAATTACCGCGATGAAGTACTCAGTTGTGCCTGTTTATCAAGAATTTGCCCGCCAAATTG	297
45	TAATTACCGCGATGAAGTACTCAGTTGTGCCTGTTTATCAAGAATTTGCCCGCCAAATTG	299
46	TAATTACCGCGATGAAGTACTCAGTTGTGCCTGTTTATCAAGAATTTGCCCGCCAAATTG	297
47	TAATTACCGCGATGAAGTACTCAGTTGTGCCTGTTTATCAAGAATTTGCCCGCCAAATTG	298
48	TAATTACCGCGATGAAGTACTCAGTTGTGCCTGTTTATCAAGAATTTGCCCGCCAAATTG	298
49	TAATTACCGCGATGAAGTACTCAGTTGTGCCTGTTTATCAAGAATTTGCCCGCCAAATTG	297
50	TAATTACCGCGATGAAGTACTCAGTTGTGCCTGTTTATCAAGAATTTGCCCGCCAAATTG	297
51	TAATCACCGCGATGAAATATTCAGTTGTGCCTGTTTATCAAGAATTTGCCCGCCAAATTG	297
52	TAATCACCGCGATGAAATATTCAGTTGTGCCTGTTTATCAAGAATTTGCCCGCCAAATTG	297
53	TAATCACCGCGATGAAATATTCAGTTGTGCCTGTTTATCAAGAATTTGCCCGCCAAATTG	297

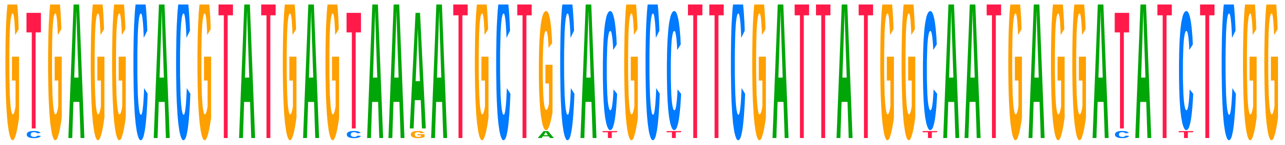

	GTGAGGCACGTATGAGTAAAATGCTGCACGCCTTCGATTATGGCAATGAGGATATCTCGG	
1	GTGAGGCACGTATGAGTAAAATGCTGCACGCCTTCGATTATGGCAATGAGGATATCTCGG	357
2	GTGAGGCACGTATGAGTAAAATGCTGCACGCCTTCGATTATGGCAATGAGGATATCTCGG	357
3	GTGAGGCACGTATGAGTAAAATGCTGCACGCCTTCGATTATGGCAATGAGGATATCTCGG	357
4	GTGAGGCACGTATGAGTAAAATGCTGCACGCCTTCGATTATGGCAATGAGGATATCTCGG	357
5	GTGAGGCACGTATGAGTAAAATGCTGCACGCCTTCGATTATGGCAATGAGGATATCTCGG	357
6	GTGAGGCACGTATGAGTAAAATGCTGCACGCCTTCGATTATGGCAATGAGGATATCTCGG	357
7	GTGAGGCACGTATGAGTAAAATGCTGCACGCCTTCGATTATGGCAATGAGGATATCTCGG	357
8	GTGAGGCACGTATGAGTAAAATGCTGCACGCCTTCGATTATGGCAATGAGGATATCTCGG	357
9	GTGAGGCACGTATGAGTAAAATGCTGCACGCCTTCGATTATGGCAATGAGGATATCTCGG	357
10	GTGAGGCACGTATGAGTAAAATGCTGCACGCCTTCGATTATGGCAATGAGGATATCTCGG	357
11	GTGAGGCACGTATGAGTAAAATGCTGCACGCCTTCGATTATGGCAATGAGGATATCTCGG	357
12	GTGAGGCACGTATGAGTAAAATGCTGCACGCCTTCGATTATGGCAATGAGGATATCTCGG	357
13	GTGAGGCACGTATGAGTAAAATGCTGCACGCCTTCGATTATGGCAATGAGGATATCTCGG	357
14	GTGAGGCACGTATGAGTAAAATGCTGCACGCCTTCGATTATGGCAATGAGGATATCTCGG	357
15	GTGAGGCACGTATGAGTAAAATGCTGCACGCCTTCGATTATGGCAATGAGGATATCTCGG	357
16	GTGAGGCACGTATGAGTAAAATGCTGCACGCCTTCGATTATGGCAATGAGGATATCTCGG	357
17	GTGAGGCACGTATGAGTAAAATGCTGCACGCCTTCGATTATGGCAATGAGGATATCTCGG	357
18	GTGAGGCACGTATGAGTAAAATGCTGCACGCCTTCGATTATGGCAATGAGGATATCTCGG	357
19	GTGAGGCACGTATGAGTAAAATGCTGCACGCCTTCGATTATGGCAATGAGGATATCTCGG	357
20	GTGAGGCACGTATGAGTAAAATGCTGCACGCCTTCGATTATGGCAATGAGGATATCTCGG	357
21	GTGAGGCACGTATGAGTAAAATGCTGCACGCCTTCGATTATGGCAATGAGGATATCTCGG	357
22	GTGAGGCACGTATGAGTAAAATGCTGCACGCCTTCGATTATGGCAATGAGGATATCTCGG	357
23	GTGAGGCACGTATGAGTAAAATGCTGCACGCCTTCGATTATGGCAATGAGGATATCTCGG	357
24	GTGAGGCACGTATGAGTAAAATGCTGCACGCCTTCGATTATGGCAATGAGGATATCTCGG	357
25	GTGAGGCACGTATGAGTAAAATGCTGCACGCCTTCGATTATGGCAATGAGGATATCTCGG	357
26	GTGAGGCACGTATGAGTAAAATGCTGCACGCCTTCGATTATGGCAATGAGGATATCTCGG	357
27	GTGAGGCACGTATGAGTAAAATGCTGCACGCCTTCGATTATGGCAATGAGGATATCTCGG	357
28	GTGAGGCACGTATGAGTAAAATGCTGCACGCCTTCGATTATGGCAATGAGGATATCTCGG	357
29	GTGAGGCACGTATGAGTAAAATGCTGCACGCCTTCGATTATGGCAATGAGGATATCTCGG	357
30	GTGAGGCACGTATGAGTAAAATGCTGCACGCCTTCGATTATGGCAATGAGGATATCTCGG	357
31	GTGAGGCACGTATGAGTAAAATGCTGCACGCCTTCGATTATGGCAATGAGGATATCTCGG	357
32	GTGAGGCACGTATGAGTAAAATGCTGCACGCCTTCGATTATGGCAATGAGGATATCTCGG	357
33	GTGAGGCACGTATGAGTAAAATGCTGCACGCCTTCGATTATGGCAATGAGGATATCTCGG	357
34	GTGAGGCACGTATGAGTAAAATGCTGCACGCCTTCGATTATGGCAATGAGGATATCTCGG	357
35	GTGAGGCACGTATGAGTAAAATGCTGCACGCCTTCGATTATGGCAATGAGGATATCTCGG	357
36	GTGAGGCACGTATGAGTAAAATGCTGCACGCCTTCGATTATGGCAATGAGGATATCTCGG	357
37	GTGAGGCACGTATGAGTAAAATGCTGCACGCCTTCGATTATGGCAATGAGGATATCTCGG	357
38	GTGAGGCACGTATGAGTAAAATGCTGCACGCCTTCGATTATGGCAATGAGGATATCTCGG	357
39	GTGAGGCACGTATGAGTAAAATGCTGCACGCCTTCGATTATGGCAATGAGGATATCTCGG	357
40	GTGAGGCACGTATGAGTAAAATGCTGCACGCCTTCGATTATGGCAATGAGGATATCTCGG	357
41	GTGAGGCACGTATGAGTAAAATGCTGCACGCCTTCGATTATGGCAATGAGGATATCTCGG	357
42	GTGAGGCACGTATGAGTAAAATGCTGCACGCCTTCGATTATGGCAATGAGGATATCTCGG	357
43	GTGAGGCACGTATGAGTAAAATGCTGCACGCCTTCGATTATGGCAATGAGGATATCTCGG	357
44	GTGAGGCACGTATGAGTAAAATGCTGCACGCCTTCGATTATGGCAATGAGGATATCTCGG	357
45	GTGAGGCACGTATGAGTAAAATGCTGCACGCCTTCGATTATGGCAATGAGGATATCTCGG	359
46	GTGAGGCACGTATGAGTAAAATGCTGCACGCCTTCGATTATGGCAATGAGGATATCTCGG	357
47	GTGAGGCACGTATGAGTAAAATGCTGCACGCCTTCGATTATGGCAATGAGGATATCTCGG	358
48	GTGAGGCACGTATGAGTAAAATGCTGCACGCCTTCGATTATGGCAATGAGGATATCTCGG	358
49	GTGAGGCACGTATGAGTAAAATGCTGCACGCCTTCGATTATGGCAATGAGGATATCTCGG	357
50	GTGAGGCACGTATGAGTAAAATGCTGCACGCCTTCGATTATGGCAATGAGGATATCTCGG	357
51	GCGAGGCACGTATGAGCAAGATGCTACATGCTTTCGATTATGGTAATGAGGACATTTCGG	357
52	GCGAGGCACGTATGAGCAAGATGCTACATGCTTTCGATTATGGTAATGAGGACATTTCGG	357
53	GCGAGGCACGTATGAGCAAGATGCTACATGCTTTCGATTATGGTAATGAGGACATTTCGG	357

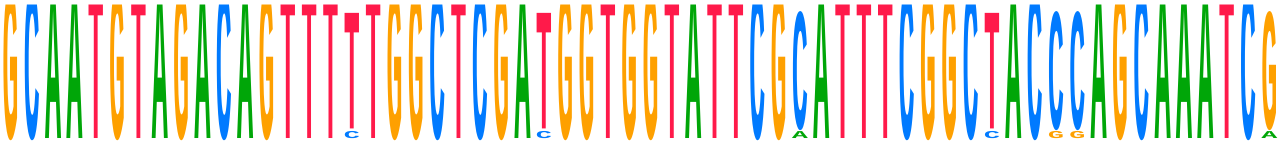

	GCAATGTAGACAGTTTTTGGCTCGATGGTGGTATTCGCATTTCGGCTACCCAGCAAATCG	
1	GCAATGTAGACAGTTTTTGGCTCGATGGTGGTATTCGCATTTCGGCTACCCAGCAAATCG	417
2	GCAATGTAGACAGTTTTTGGCTCGATGGTGGTATTCGCATTTCGGCTACCCAGCAAATCG	417
3	GCAATGTAGACAGTTTTTGGCTCGATGGTGGTATTCGCATTTCGGCTACCCAGCAAATCG	417
4	GCAATGTAGACAGTTTTTGGCTCGATGGTGGTATTCGCATTTCGGCTACCCAGCAAATCG	417
5	GCAATGTAGACAGTTTTTGGCTCGATGGTGGTATTCGCATTTCGGCTACCCAGCAAATCG	417
6	GCAATGTAGACAGTTTTTGGCTCGATGGTGGTATTCGCATTTCGGCTACCCAGCAAATCG	417
7	GCAATGTAGACAGTTTTTGGCTCGATGGTGGTATTCGCATTTCGGCTACCCAGCAAATCG	417
8	GCAATGTAGACAGTTTTTGGCTCGATGGTGGTATTCGCATTTCGGCTACCCAGCAAATCG	417
9	GCAATGTAGACAGTTTTTGGCTCGATGGTGGTATTCGCATTTCGGCTACCCAGCAAATCG	417
10	GCAATGTAGACAGTTTTTGGCTCGATGGTGGTATTCGCATTTCGGCTACCCAGCAAATCG	417
11	GCAATGTAGACAGTTTTTGGCTCGATGGTGGTATTCGCATTTCGGCTACCCAGCAAATCG	417
12	GCAATGTAGACAGTTTTTGGCTCGATGGTGGTATTCGCATTTCGGCTACCCAGCAAATCG	417
13	GCAATGTAGACAGTTTTTGGCTCGATGGTGGTATTCGCATTTCGGCTACCCAGCAAATCG	417
14	GCAATGTAGACAGTTTTTGGCTCGATGGTGGTATTCGCATTTCGGCTACCCAGCAAATCG	417
15	GCAATGTAGACAGTTTTTGGCTCGATGGTGGTATTCGCATTTCGGCTACCCAGCAAATCG	417
16	GCAATGTAGACAGTTTTTGGCTCGATGGTGGTATTCGCATTTCGGCTACCCAGCAAATCG	417
17	GCAATGTAGACAGTTTTTGGCTCGATGGTGGTATTCGCATTTCGGCTACCCAGCAAATCG	417
18	GCAATGTAGACAGTTTTTGGCTCGATGGTGGTATTCGCATTTCGGCTACCCAGCAAATCG	417
19	GCAATGTAGACAGTTTTTGGCTCGATGGTGGTATTCGCATTTCGGCTACCCAGCAAATCG	417
20	GCAATGTAGACAGTTTTTGGCTCGATGGTGGTATTCGCATTTCGGCTACCCAGCAAATCG	417
21	GCAATGTAGACAGTTTTTGGCTCGATGGTGGTATTCGCATTTCGGCTACCCAGCAAATCG	417
22	GCAATGTAGACAGTTTTTGGCTCGATGGTGGTATTCGCATTTCGGCTACCCAGCAAATCG	417
23	GCAATGTAGACAGTTTTTGGCTCGATGGTGGTATTCGCATTTCGGCTACCCAGCAAATCG	417
24	GCAATGTAGACAGTTTTTGGCTCGATGGTGGTATTCGCATTTCGGCTACCCAGCAAATCG	417
25	GCAATGTAGACAGTTTTTGGCTCGATGGTGGTATTCGCATTTCGGCTACCCAGCAAATCG	417
26	GCAATGTAGACAGTTTTTGGCTCGATGGTGGTATTCGCATTTCGGCTACCCAGCAAATCG	417
27	GCAATGTAGACAGTTTTTGGCTCGATGGTGGTATTCGCATTTCGGCTACCCAGCAAATCG	417
28	GCAATGTAGACAGTTTTTGGCTCGATGGTGGTATTCGCATTTCGGCTACCCAGCAAATCG	417
29	GCAATGTAGACAGTTTTTGGCTCGATGGTGGTATTCGCATTTCGGCTACCCAGCAAATCG	417
30	GCAATGTAGACAGTTTTTGGCTCGATGGTGGTATTCGCATTTCGGCTACCCAGCAAATCG	417
31	GCAATGTAGACAGTTTTTGGCTCGATGGTGGTATTCGCATTTCGGCTACCCAGCAAATCG	417
32	GCAATGTAGACAGTTTTTGGCTCGATGGTGGTATTCGCATTTCGGCTACCCAGCAAATCG	417
33	GCAATGTAGACAGTTTTTGGCTCGATGGTGGTATTCGCATTTCGGCTACCCAGCAAATCG	417
34	GCAATGTAGACAGTTTTTGGCTCGATGGTGGTATTCGCATTTCGGCTACCCAGCAAATCG	417
35	GCAATGTAGACAGTTTTTGGCTCGATGGTGGTATTCGCATTTCGGCTACCCAGCAAATCG	417
36	GCAATGTAGACAGTTTTTGGCTCGATGGTGGTATTCGCATTTCGGCTACCCAGCAAATCG	417
37	GCAATGTAGACAGTTTTTGGCTCGATGGTGGTATTCGCATTTCGGCTACCCAGCAAATCG	417
38	GCAATGTAGACAGTTTTTGGCTCGATGGTGGTATTCGCATTTCGGCTACCCAGCAAATCG	417
39	GCAATGTAGACAGTTTTTGGCTCGATGGTGGTATTCGCATTTCGGCTACCCAGCAAATCG	417
40	GCAATGTAGACAGTTTTTGGCTCGATGGTGGTATTCGCATTTCGGCTACCCAGCAAATCG	417
41	GCAATGTAGACAGTTTTTGGCTCGATGGTGGTATTCGCATTTCGGCTACCCAGCAAATCG	417
42	GCAATGTAGACAGTTTTTGGCTCGATGGTGGTATTCGCATTTCGGCTACCCAGCAAATCG	417
43	GCAATGTAGACAGTTTTTGGCTCGATGGTGGTATTCGCATTTCGGCTACCCAGCAAATCG	417
44	GCAATGTAGACAGTTTTTGGCTCGATGGTGGTATTCGCATTTCGGCTACCCAGCAAATCG	417
45	GCAATGTAGACAGTTTTTGGCTCGATGGTGGTATTCGCATTTCGGCTACCCAGCAAATCG	419
46	GCAATGTAGACAGTTTTTGGCTCGATGGTGGTATTCGCATTTCGGCTACCCAGCAAATCG	417
47	GCAATGTAGACAGTTTTTGGCTCGATGGTGGTATTCGCATTTCGGCTACCCAGCAAATCG	418
48	GCAATGTAGACAGTTTTTGGCTCGATGGTGGTATTCGCATTTCGGCTACCCAGCAAATCG	418
49	GCAATGTAGACAGTTTTTGGCTCGATGGTGGTATTCGCATTTCGGCTACCCAGCAAATCG	417
50	GCAATGTAGACAGTTTTTGGCTCGATGGTGGTATTCGCATTTCGGCTACCCAGCAAATCG	417
51	GCAATGTAGACAGTTTCTGGCTCGACGGTGGTATTCGAATTTCGGCCACGGAGCAAATCA	417
52	GCAATGTAGACAGTTTCTGGCTCGACGGTGGTATTCGAATTTCGGCCACGGAGCAAATCA	417
53	GCAATGTAGACAGTTTCTGGCTCGACGGTGGTATTCGAATTTCGGCCACGGAGCAAATCA	417

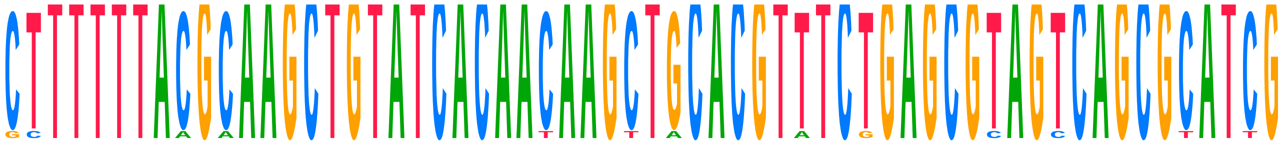

CTTTTTTACGCAAGCTGTATCACAACAAGCTGCACGTTTCTGAGCGTAGTCAGCGCATCG

1	CTTTTTTACGCAAGCTGTATCACAACAAGCTGCACGTTTCTGAGCGTAGTCAGCGCATCG	477
2	CTTTTTTACGCAAGCTGTATCACAACAAGCTGCACGTTTCTGAGCGTAGTCAGCGCATCG	477
3	CTTTTTTACGCAAGCTGTATCACAACAAGCTGCACGTTTCTGAGCGTAGTCAGCGCATCG	477
4	CTTTTTTACGCAAGCTGTATCACAACAAGCTGCACGTTTCTGAGCGTAGTCAGCGCATCG	477
5	CTTTTTTACGCAAGCTGTATCACAACAAGCTGCACGTTTCTGAGCGTAGTCAGCGCATCG	477
6	CTTTTTTACGCAAGCTGTATCACAACAAGCTGCACGTTTCTGAGCGTAGTCAGCGCATCG	477
7	CTTTTTTACGCAAGCTGTATCACAACAAGCTGCACGTTTCTGAGCGTAGTCAGCGCATCG	477
8	CTTTTTTACGCAAGCTGTATCACAACAAGCTGCACGTTTCTGAGCGTAGTCAGCGCATCG	477
9	CTTTTTTACGCAAGCTGTATCACAACAAGCTGCACGTTTCTGAGCGTAGTCAGCGCATCG	477
10	CTTTTTTACGCAAGCTGTATCACAACAAGCTGCACGTTTCTGAGCGTAGTCAGCGCATCG	477
11	CTTTTTTACGCAAGCTGTATCACAACAAGCTGCACGTTTCTGAGCGTAGTCAGCGCATCG	477
12	CTTTTTTACGCAAGCTGTATCACAACAAGCTGCACGTTTCTGAGCGTAGTCAGCGCATCG	477
13	CTTTTTTACGCAAGCTGTATCACAACAAGCTGCACGTTTCTGAGCGTAGTCAGCGCATCG	477
14	CTTTTTTACGCAAGCTGTATCACAACAAGCTGCACGTTTCTGAGCGTAGTCAGCGCATCG	477
15	CTTTTTTACGCAAGCTGTATCACAACAAGCTGCACGTTTCTGAGCGTAGTCAGCGCATCG	477
16	CTTTTTTACGCAAGCTGTATCACAACAAGCTGCACGTTTCTGAGCGTAGTCAGCGCATCG	477
17	CTTTTTTACGCAAGCTGTATCACAACAAGCTGCACGTTTCTGAGCGTAGTCAGCGCATCG	477
18	CTTTTTTACGCAAGCTGTATCACAACAAGCTGCACGTTTCTGAGCGTAGTCAGCGCATCG	477
19	CTTTTTTACGCAAGCTGTATCACAACAAGCTGCACGTTTCTGAGCGTAGTCAGCGCATCG	477
20	CTTTTTTACGCAAGCTGTATCACAACAAGCTGCACGTTTCTGAGCGTAGTCAGCGCATCG	477
21	CTTTTTTACGCAAGCTGTATCACAACAAGCTGCACGTTTCTGAGCGTAGTCAGCGCATCG	477
22	CTTTTTTACGCAAGCTGTATCACAACAAGCTGCACGTTTCTGAGCGTAGTCAGCGCATCG	477
23	CTTTTTTACGCAAGCTGTATCACAACAAGCTGCACGTTTCTGAGCGTAGTCAGCGCATCG	477
24	CTTTTTTACGCAAGCTGTATCACAACAAGCTGCACGTTTCTGAGCGTAGTCAGCGCATCG	477
25	CTTTTTTACGCAAGCTGTATCACAACAAGCTGCACGTTTCTGAGCGTAGTCAGCGCATCG	477
26	CTTTTTTACGCAAGCTGTATCACAACAAGCTGCACGTTTCTGAGCGTAGTCAGCGCATCG	477
27	CTTTTTTACGCAAGCTGTATCACAACAAGCTGCACGTTTCTGAGCGTAGTCAGCGCATCG	477
28	CTTTTTTACGCAAGCTGTATCACAACAAGCTGCACGTTTCTGAGCGTAGTCAGCGCATCG	477
29	CTTTTTTACGCAAGCTGTATCACAACAAGCTGCACGTTTCTGAGCGTAGTCAGCGCATCG	477
30	CTTTTTTACGCAAGCTGTATCACAACAAGCTGCACGTTTCTGAGCGTAGTCAGCGCATCG	477
31	CTTTTTTACGCAAGCTGTATCACAACAAGCTGCACGTTTCTGAGCGTAGTCAGCGCATCG	477
32	CTTTTTTACGCAAGCTGTATCACAACAAGCTGCACGTTTCTGAGCGTAGTCAGCGCATCG	477
33	CTTTTTTACGCAAGCTGTATCACAACAAGCTGCACGTTTCTGAGCGTAGTCAGCGCATCG	477
34	CTTTTTTACGCAAGCTGTATCACAACAAGCTGCACGTTTCTGAGCGTAGTCAGCGCATCG	477
35	CTTTTTTACGCAAGCTGTATCACAACAAGCTGCACGTTTCTGAGCGTAGTCAGCGCATCG	477
36	CTTTTTTACGCAAGCTGTATCACAACAAGCTGCACGTTTCTGAGCGTAGTCAGCGCATCG	477
37	CTTTTTTACGCAAGCTGTATCACAACAAGCTGCACGTTTCTGAGCGTAGTCAGCGCATCG	477
38	CTTTTTTACGCAAGCTGTATCACAACAAGCTGCACGTTTCTGAGCGTAGTCAGCGCATCG	477
39	CTTTTTTACGCAAGCTGTATCACAACAAGCTGCACGTTTCTGAGCGTAGTCAGCGCATCG	477
40	CTTTTTTACGCAAGCTGTATCACAACAAGCTGCACGTTTCTGAGCGTAGTCAGCGCATCG	477
41	CTTTTTTACGCAAGCTGTATCACAACAAGCTGCACGTTTCTGAGCGTAGTCAGCGCATCG	477
42	CTTTTTTACGCAAGCTGTATCACAACAAGCTGCACGTTTCTGAGCGTAGTCAGCGCATCG	477
43	CTTTTTTACGCAAGCTGTATCACAACAAGCTGCACGTTTCTGAGCGTAGTCAGCGCATCG	477
44	CTTTTTTACGCAAGCTGTATCACAACAAGCTGCACGTTTCTGAGCGTAGTCAGCGCATCG	477
45	CTTTTTTACGCAAGCTGTATCACAACAAGCTGCACGTTTCTGAGCGTAGTCAGCGCATCG	479
46	CTTTTTTACGCAAGCTGTATCACAACAAGCTGCACGTTTCTGAGCGTAGTCAGCGCATCG	477
47	CTTTTTTACGCAAGCTGTATCACAACAAGCTGCACGTTTCTGAGCGTAGTCAGCGCATCG	478
48	CTTTTTTACGCAAGCTGTATCACAACAAGCTGCACGTTTCTGAGCGTAGTCAGCGCATCG	478
49	CTTTTTTACGCAAGCTGTATCACAACAAGCTGCACGTTTCTGAGCGTAGTCAGCGCATCG	477
50	CTTTTTTACGCAAGCTGTATCACAACAAGCTGCACGTTTCTGAGCGTAGTCAGCGCATCG	477
51	GCTTTTTAAGAAAGCTGTATCACAATAAGTTACACGTATCGGAGCGCAGCCAGCGTATTG	477
52	GCTTTTTAAGAAAGCTGTATCACAATAAGTTACACGTATCGGAGCGCAGCCAGCGTATTG	477
53	GCTTTTTAAGAAAGCTGTATCACAATAAGTTACACGTATCGGAGCGCAGCCAGCGTATTG	477

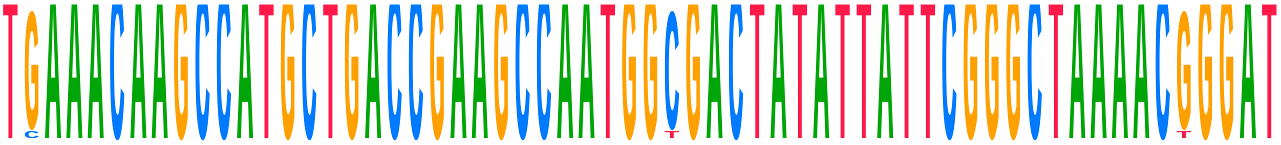

TGAAACAAGCCATGCTGACCGAAGCCAATGGCGACTATATTATTCGGGCTAAAACGGGAT

1	TGAAACAAGCCATGCTGACCGAAGCCAATGGCGACTATATTATTCGGGCTAAAACGGGAT	537
2	TGAAACAAGCCATGCTGACCGAAGCCAATGGCGACTATATTATTCGGGCTAAAACGGGAT	537
3	TGAAACAAGCCATGCTGACCGAAGCCAATGGCGACTATATTATTCGGGCTAAAACGGGAT	537
4	TGAAACAAGCCATGCTGACCGAAGCCAATGGCGACTATATTATTCGGGCTAAAACGGGAT	537
5	TGAAACAAGCCATGCTGACCGAAGCCAATGGCGACTATATTATTCGGGCTAAAACGGGAT	537
6	TGAAACAAGCCATGCTGACCGAAGCCAATGGCGACTATATTATTCGGGCTAAAACGGGAT	537
7	TGAAACAAGCCATGCTGACCGAAGCCAATGGCGACTATATTATTCGGGCTAAAACGGGAT	537
8	TGAAACAAGCCATGCTGACCGAAGCCAATGGCGACTATATTATTCGGGCTAAAACGGGAT	537
9	TGAAACAAGCCATGCTGACCGAAGCCAATGGCGACTATATTATTCGGGCTAAAACGGGAT	537
10	TGAAACAAGCCATGCTGACCGAAGCCAATGGCGACTATATTATTCGGGCTAAAACGGGAT	537
11	TGAAACAAGCCATGCTGACCGAAGCCAATGGCGACTATATTATTCGGGCTAAAACGGGAT	537
12	TGAAACAAGCCATGCTGACCGAAGCCAATGGCGACTATATTATTCGGGCTAAAACGGGAT	537
13	TGAAACAAGCCATGCTGACCGAAGCCAATGGCGACTATATTATTCGGGCTAAAACGGGAT	537
14	TGAAACAAGCCATGCTGACCGAAGCCAATGGCGACTATATTATTCGGGCTAAAACGGGAT	537
15	TGAAACAAGCCATGCTGACCGAAGCCAATGGCGACTATATTATTCGGGCTAAAACGGGAT	537
16	TGAAACAAGCCATGCTGACCGAAGCCAATGGCGACTATATTATTCGGGCTAAAACGGGAT	537
17	TGAAACAAGCCATGCTGACCGAAGCCAATGGCGACTATATTATTCGGGCTAAAACGGGAT	537
18	TGAAACAAGCCATGCTGACCGAAGCCAATGGCGACTATATTATTCGGGCTAAAACGGGAT	537
19	TGAAACAAGCCATGCTGACCGAAGCCAATGGCGACTATATTATTCGGGCTAAAACGGGAT	537
20	TGAAACAAGCCATGCTGACCGAAGCCAATGGCGACTATATTATTCGGGCTAAAACGGGAT	537
21	TGAAACAAGCCATGCTGACCGAAGCCAATGGCGACTATATTATTCGGGCTAAAACGGGAT	537
22	TGAAACAAGCCATGCTGACCGAAGCCAATGGCGACTATATTATTCGGGCTAAAACGGGAT	537
23	TGAAACAAGCCATGCTGACCGAAGCCAATGGCGACTATATTATTCGGGCTAAAACGGGAT	537
24	TGAAACAAGCCATGCTGACCGAAGCCAATGGCGACTATATTATTCGGGCTAAAACGGGAT	537
25	TGAAACAAGCCATGCTGACCGAAGCCAATGGCGACTATATTATTCGGGCTAAAACGGGAT	537
26	TGAAACAAGCCATGCTGACCGAAGCCAATGGCGACTATATTATTCGGGCTAAAACGGGAT	537
27	TGAAACAAGCCATGCTGACCGAAGCCAATGGCGACTATATTATTCGGGCTAAAACGGGAT	537
28	TGAAACAAGCCATGCTGACCGAAGCCAATGGCGACTATATTATTCGGGCTAAAACGGGAT	537
29	TGAAACAAGCCATGCTGACCGAAGCCAATGGCGACTATATTATTCGGGCTAAAACGGGAT	537
30	TGAAACAAGCCATGCTGACCGAAGCCAATGGCGACTATATTATTCGGGCTAAAACGGGAT	537
31	TGAAACAAGCCATGCTGACCGAAGCCAATGGCGACTATATTATTCGGGCTAAAACGGGAT	537
32	TGAAACAAGCCATGCTGACCGAAGCCAATGGCGACTATATTATTCGGGCTAAAACGGGAT	537
33	TGAAACAAGCCATGCTGACCGAAGCCAATGGCGACTATATTATTCGGGCTAAAACGGGAT	537
34	TGAAACAAGCCATGCTGACCGAAGCCAATGGCGACTATATTATTCGGGCTAAAACGGGAT	537
35	TGAAACAAGCCATGCTGACCGAAGCCAATGGCGACTATATTATTCGGGCTAAAACGGGAT	537
36	TGAAACAAGCCATGCTGACCGAAGCCAATGGCGACTATATTATTCGGGCTAAAACGGGAT	537
37	TGAAACAAGCCATGCTGACCGAAGCCAATGGCGACTATATTATTCGGGCTAAAACGGGAT	537
38	TGAAACAAGCCATGCTGACCGAAGCCAATGGCGACTATATTATTCGGGCTAAAACGGGAT	537
39	TGAAACAAGCCATGCTGACCGAAGCCAATGGCGACTATATTATTCGGGCTAAAACGGGAT	537
40	TGAAACAAGCCATGCTGACCGAAGCCAATGGCGACTATATTATTCGGGCTAAAACGGGAT	537
41	TGAAACAAGCCATGCTGACCGAAGCCAATGGCGACTATATTATTCGGGCTAAAACGGGAT	537
42	TGAAACAAGCCATGCTGACCGAAGCCAATGGCGACTATATTATTCGGGCTAAAACGGGAT	537
43	TGAAACAAGCCATGCTGACCGAAGCCAATGGCGACTATATTATTCGGGCTAAAACGGGAT	537
44	TGAAACAAGCCATGCTGACCGAAGCCAATGGCGACTATATTATTCGGGCTAAAACGGGAT	537
45	TGAAACAAGCCATGCTGACCGAAGCCAATGGCGACTATATTATTCGGGCTAAAACGGGAT	539
46	TGAAACAAGCCATGCTGACCGAAGCCAATGGCGACTATATTATTCGGGCTAAAACGGGAT	537
47	TGAAACAAGCCATGCTGACCGAAGCCAATGGCGACTATATTATTCGGGCTAAAACGGGAT	538
48	TGAAACAAGCCATGCTGACCGAAGCCAATGGCGACTATATTATTCGGGCTAAAACGGGAT	538
49	TGAAACAAGCCATGCTGACCGAAGCCAATGGCGACTATATTATTCGGGCTAAAACGGGAT	537
50	TGAAACAAGCCATGCTGACCGAAGCCAATGGCGACTATATTATTCGGGCTAAAACGGGAT	537
51	TCAAACAAGCCATGCTGACCGAAGCCAATGGTGACTATATTATTCGGGCTAAAACTGGAT	537
52	TCAAACAAGCCATGCTGACCGAAGCCAATGGTGACTATATTATTCGGGCTAAAACTGGAT	537
53	TCAAACAAGCCATGCTGACCGAAGCCAATGGTGACTATATTATTCGGGCTAAAACTGGAT	537

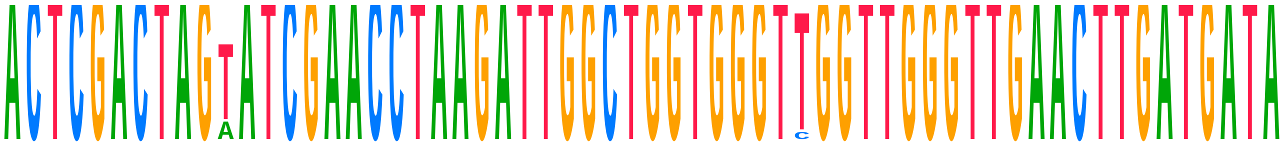

	ACTCGACTAGTATCGAACCTAAGATTGGCTGGTGGGTTGGTTGGGTTGAACTTGATGATA	
1	ACTCGACTAGTATCGAACCTAAGATTGGCTGGTGGGTTGGTTGGGTTGAACTTGATGATA	597
2	ACTCGACTAGTATCGAACCTAAGATTGGCTGGTGGGTTGGTTGGGTTGAACTTGATGATA	597
3	ACTCGACTAGTATCGAACCTAAGATTGGCTGGTGGGTTGGTTGGGTTGAACTTGATGATA	597
4	ACTCGACTAGTATCGAACCTAAGATTGGCTGGTGGGTTGGTTGGGTTGAACTTGATGATA	597
5	ACTCGACTAGTATCGAACCTAAGATTGGCTGGTGGGTTGGTTGGGTTGAACTTGATGATA	597
6	ACTCGACTAGTATCGAACCTAAGATTGGCTGGTGGGTTGGTTGGGTTGAACTTGATGATA	597
7	ACTCGACTAGTATCGAACCTAAGATTGGCTGGTGGGTTGGTTGGGTTGAACTTGATGATA	597
8	ACTCGACTAGTATCGAACCTAAGATTGGCTGGTGGGTTGGTTGGGTTGAACTTGATGATA	597
9	ACTCGACTAGTATCGAACCTAAGATTGGCTGGTGGGTTGGTTGGGTTGAACTTGATGATA	597
10	ACTCGACTAGTATCGAACCTAAGATTGGCTGGTGGGTTGGTTGGGTTGAACTTGATGATA	597
11	ACTCGACTAGTATCGAACCTAAGATTGGCTGGTGGGTTGGTTGGGTTGAACTTGATGATA	597
12	ACTCGACTAGTATCGAACCTAAGATTGGCTGGTGGGTTGGTTGGGTTGAACTTGATGATA	597
13	ACTCGACTAGTATCGAACCTAAGATTGGCTGGTGGGTTGGTTGGGTTGAACTTGATGATA	597
14	ACTCGACTAGTATCGAACCTAAGATTGGCTGGTGGGTTGGTTGGGTTGAACTTGATGATA	597
15	ACTCGACTAGTATCGAACCTAAGATTGGCTGGTGGGTTGGTTGGGTTGAACTTGATGATA	597
16	ACTCGACTAGTATCGAACCTAAGATTGGCTGGTGGGTTGGTTGGGTTGAACTTGATGATA	597
17	ACTCGACTAGTATCGAACCTAAGATTGGCTGGTGGGTTGGTTGGGTTGAACTTGATGATA	597
18	ACTCGACTAGTATCGAACCTAAGATTGGCTGGTGGGTTGGTTGGGTTGAACTTGATGATA	597
19	ACTCGACTAGTATCGAACCTAAGATTGGCTGGTGGGTTGGTTGGGTTGAACTTGATGATA	597
20	ACTCGACTAGTATCGAACCTAAGATTGGCTGGTGGGTTGGTTGGGTTGAACTTGATGATA	597
21	ACTCGACTAGTATCGAACCTAAGATTGGCTGGTGGGTTGGTTGGGTTGAACTTGATGATA	597
22	ACTCGACTAGTATCGAACCTAAGATTGGCTGGTGGGTTGGTTGGGTTGAACTTGATGATA	597
23	ACTCGACTAGTATCGAACCTAAGATTGGCTGGTGGGTTGGTTGGGTTGAACTTGATGATA	597
24	ACTCGACTAGTATCGAACCTAAGATTGGCTGGTGGGTTGGTTGGGTTGAACTTGATGATA	597
25	ACTCGACTAGTATCGAACCTAAGATTGGCTGGTGGGTTGGTTGGGTTGAACTTGATGATA	597
26	ACTCGACTAGTATCGAACCTAAGATTGGCTGGTGGGTTGGTTGGGTTGAACTTGATGATA	597
27	ACTCGACTAGTATCGAACCTAAGATTGGCTGGTGGGTTGGTTGGGTTGAACTTGATGATA	597
28	ACTCGACTAGTATCGAACCTAAGATTGGCTGGTGGGTTGGTTGGGTTGAACTTGATGATA	597
29	ACTCGACTAGTATCGAACCTAAGATTGGCTGGTGGGTTGGTTGGGTTGAACTTGATGATA	597
30	ACTCGACTAGTATCGAACCTAAGATTGGCTGGTGGGTTGGTTGGGTTGAACTTGATGATA	597
31	ACTCGACTAGAATCGAACCTAAGATTGGCTGGTGGGTTGGTTGGGTTGAACTTGATGATA	597
32	ACTCGACTAGTATCGAACCTAAGATTGGCTGGTGGGTTGGTTGGGTTGAACTTGATGATA	597
33	ACTCGACTAGAATCGAACCTAAGATTGGCTGGTGGGTTGGTTGGGTTGAACTTGATGATA	597
34	ACTCGACTAGAATCGAACCTAAGATTGGCTGGTGGGTTGGTTGGGTTGAACTTGATGATA	597
35	ACTCGACTAGTATCGAACCTAAGATTGGCTGGTGGGTTGGTTGGGTTGAACTTGATGATA	597
36	ACTCGACTAGTATCGAACCTAAGATTGGCTGGTGGGTTGGTTGGGTTGAACTTGATGATA	597
37	ACTCGACTAGTATCGAACCTAAGATTGGCTGGTGGGTTGGTTGGGTTGAACTTGATGATA	597
38	ACTCGACTAGAATCGAACCTAAGATTGGCTGGTGGGTTGGTTGGGTTGAACTTGATGATA	597
39	ACTCGACTAGTATCGAACCTAAGATTGGCTGGTGGGTTGGTTGGGTTGAACTTGATGATA	597
40	ACTCGACTAGTATCGAACCTAAGATTGGCTGGTGGGTTGGTTGGGTTGAACTTGATGATA	597
41	ACTCGACTAGAATCGAACCTAAGATTGGCTGGTGGGTTGGTTGGGTTGAACTTGATGATA	597
42	ACTCGACTAGTATCGAACCTAAGATTGGCTGGTGGGTTGGTTGGGTTGAACTTGATGATA	597
43	ACTCGACTAGAATCGAACCTAAGATTGGCTGGTGGGTTGGTTGGGTTGAACTTGATGATA	597
44	ACTCGACTAGTATCGAACCTAAGATTGGCTGGTGGGTTGGTTGGGTTGAACTTGATGATA	597
45	ACTCGACTAGTATCGAACCTAAGATTGGCTGGTGGGTTGGTTGGGTTGAACTTGATGATA	599
46	ACTCGACTAGTATCGAACCTAAGATTGGCTGGTGGGTTGGTTGGGTTGAACTTGATGATA	597
47	ACTCGACTAGTATCGAACCTAAGATTGGCTGGTGGGTTGGTTGGGTTGAACTTGATGATA	598
48	ACTCGACTAGAATCGAACCTAAGATTGGCTGGTGGGTTGGTTGGGTTGAACTTGATGATA	598
49	ACTCGACTAGTATCGAACCTAAGATTGGCTGGTGGGTTGGTTGGGTTGAACTTGATGATA	597
50	ACTCGACTAGTATCGAACCTAAGATTGGCTGGTGGGTTGGTTGGGTTGAACTTGATGATA	597
51	ACTCGACTAGAATCGAACCTAAGATTGGCTGGTGGGTCGGTTGGGTTGAACTTGATGATA	597
52	ACTCGACTAGAATCGAACCTAAGATTGGCTGGTGGGTCGGTTGGGTTGAACTTGATGATA	597
53	ACTCGACTAGAATCGAACCTAAGATTGGCTGGTGGGTCGGTTGGGTTGAACTTGATGATA	597

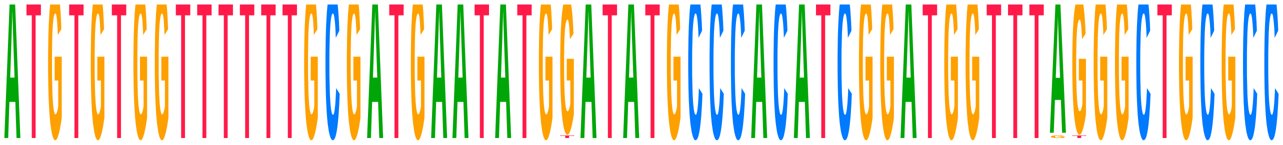

	ATGTGTGGTTTTTTGCGATGAATATGGATATGCCCACATCGGATGGTTTAGGGCTGCGCC	
1	ATGTGTGGTTTTTTGCGATGAATATGGATATGCCCACATCGGATGGTTTAGGGCTGCGCC	657
2	ATGTGTGGTTTTTTGCGATGAATATGGATATGCCCACATCGGATGGTTTAGGGCTGCGCC	657
3	ATGTGTGGTTTTTTGCGATGAATATGGATATGCCCACATCGGATGGTTTAGGGCTGCGCC	657
4	ATGTGTGGTTTTTTGCGATGAATATGGATATGCCCACATCGGATGGTTTAGGGCTGCGCC	657
5	ATGTGTGGTTTTTTGCGATGAATATGGATATGCCCACATCGGATGGTTTAGGGCTGCGCC	657
6	ATGTGTGGTTTTTTGCGATGAATATGGATATGCCCACATCGGATGGTTTAGGGCTGCGCC	657
7	ATGTGTGGTTTTTTGCGATGAATATGGATATGCCCACATCGGATGGTTTAGGGCTGCGCC	657
8	ATGTGTGGTTTTTTGCGATGAATATGGATATGCCCACATCGGATGGTTTAGGGCTGCGCC	657
9	ATGTGTGGTTTTTTGCGATGAATATGGATATGCCCACATCGGATGGTTTAGGGCTGCGCC	657
10	ATGTGTGGTTTTTTGCGATGAATATGGATATGCCCACATCGGATGGTTTAGGGCTGCGCC	657
11	ATGTGTGGTTTTTTGCGATGAATATGGATATGCCCACATCGGATGGTTTAGGGCTGCGCC	657
12	ATGTGTGGTTTTTTGCGATGAATATGGATATGCCCACATCGGATGGTTTAGGGCTGCGCC	657
13	ATGTGTGGTTTTTTGCGATGAATATGGATATGCCCACATCGGATGGTTTAGGGCTGCGCC	657
14	ATGTGTGGTTTTTTGCGATGAATATGGATATGCCCACATCGGATGGTTTAGGGCTGCGCC	657
15	ATGTGTGGTTTTTTGCGATGAATATGGATATGCCCACATCGGATGGTTTAGGGCTGCGCC	657
16	ATGTGTGGTTTTTTGCGATGAATATGGATATGCCCACATCGGATGGTTTAGGGCTGCGCC	657
17	ATGTGTGGTTTTTTGCGATGAATATGGATATGCCCACATCGGATGGTTTAGGGCTGCGCC	657
18	ATGTGTGGTTTTTTGCGATGAATATGGATATGCCCACATCGGATGGTTTAGGGCTGCGCC	657
19	ATGTGTGGTTTTTTGCGATGAATATGGATATGCCCACATCGGATGGTTTAGGGCTGCGCC	657
20	ATGTGTGGTTTTTTGCGATGAATATGGATATGCCCACATCGGATGGTTTAGGGCTGCGCC	657
21	ATGTGTGGTTTTTTGCGATGAATATGGATATGCCCACATCGGATGGTTTAGGGCTGCGCC	657
22	ATGTGTGGTTTTTTGCGATGAATATGGATATGCCCACATCGGATGGTTTAGGGCTGCGCC	657
23	ATGTGTGGTTTTTTGCGATGAATATGGATATGCCCACATCGGATGGTTTAGGGCTGCGCC	657
24	ATGTGTGGTTTTTTGCGATGAATATGGATATGCCCACATCGGATGGTTTAGGGCTGCGCC	657
25	ATGTGTGGTTTTTTGCGATGAATATGGATATGCCCACATCGGATGGTTTAGGGCTGCGCC	657
26	ATGTGTGGTTTTTTGCGATGAATATGGATATGCCCACATCGGATGGTTTAGGGCTGCGCC	657
27	ATGTGTGGTTTTTTGCGATGAATATGGATATGCCCACATCGGATGGTTTAGGGCTGCGCC	657
28	ATGTGTGGTTTTTTGCGATGAATATGGATATGCCCACATCGGATGGTTTAGGGCTGCGCC	657
29	ATGTGTGGTTTTTTGCGATGAATATGGATATGCCCACATCGGATGGTTTAGGGCTGCGCC	657
30	ATGTGTGGTTTTTTGCGATGAATATGGATATGCCCACATCGGATGGTTTAGGGCTGCGCC	657
31	ATGTGTGGTTTTTTGCGATGAATATGGATATGCCCACATCGGATGGTTTAGGGCTGCGCC	657
32	ATGTGTGGTTTTTTGCGATGAATATGGATATGCCCACATCGGATGGTTTAGGGCTGCGCC	657
33	ATGTGTGGTTTTTTGCGATGAATATGGATATGCCCACATCGGATGGTTTAGGGCTGCGCC	657
34	ATGTGTGGTTTTTTGCGATGAATATGGATATGCCCACATCGGATGGTTTAGGGCTGCGCC	657
35	ATGTGTGGTTTTTTGCGATGAATATGGATATGCCCACATCGGATGGTTTAGGGCTGCGCC	657
36	ATGTGTGGTTTTTTGCGATGAATATGGATATGCCCACATCGGATGGTTTAGGGCTGCGCC	657
37	ATGTGTGGTTTTTTGCGATGAATATGGATATGCCCACATCGGATGGTTTAGGGCTGCGCC	657
38	ATGTGTGGTTTTTTGCGATGAATATGGATATGCCCACATCGGATGGTTTAGGGCTGCGCC	657
39	ATGTGTGGTTTTTTGCGATGAATATGGATATGCCCACATCGGATGGTTTAGGGCTGCGCC	657
40	ATGTGTGGTTTTTTGCGATGAATATGGATATGCCCACATCGGATGGTTTAGGGCTGCGCC	657
41	ATGTGTGGTTTTTTGCGATGAATATGGATATGCCCACATCGGATGGTTTAGGGCTGCGCC	657
42	ATGTGTGGTTTTTTGCGATGAATATGGATATGCCCACATCGGATGGTTTAGGGCTGCGCC	657
43	ATGTGTGGTTTTTTGCGATGAATATGGATATGCCCACATCGGATGGTTTAGGGCTGCGCC	657
44	ATGTGTGGTTTTTTGCGATGAATATGGATATGCCCACATCGGATGGTTTAGGGCTGCGCC	657
45	ATGTGTGGTTTTTTGCGATGAATATGGATATGCCCACATCGGATGGTTTAGGGCTGCGCC	659
46	ATGTGTGGTTTTTTGCGATGAATATGGATATGCCCACATCGGATGGTTTAGGGCTGCGCC	657
47	ATGTGTGGTTTTTTGCGATGAATATGGATATGCCCACATCGGATGGTTTAGGGCTGCGCC	658
48	ATGTGTGGTTTTTTGCGATGAATATGGATATGCCCACATCGGATGGTTTAGGGCTGCGCC	658
49	ATGTGTGGTTTTTTGCGATGAATATGGATATGCCCACATCGGATGGTTTAGGGCTGCGCC	657
50	ATGTGTGGTTTTTTGCGATGAATATGTATATGCCCACATCGGATGGTTTGTGGCTGCGCC	657
51	ATGTGTGGTTTTTTGCGATGAATATGGATATGCCCACATCGGATGGTTTAGGGCTGCGCC	657
52	ATGTGTGGTTTTTTGCGATGAATATGGATATGCCCACATCGGATGGTTTAGGGCTGCGCC	657
53	ATGTGTGGTTTTTTGCGATGAATATGGATATGCCCACATCGGATGGTTTAGGGCTGCGCC	657

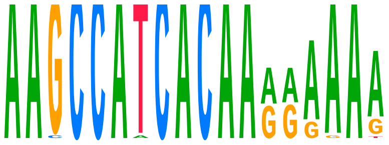

	AAGCCATCACAAAAAAAA	
1	AAGCCATCACAAGAGAAA	675
2	AAGCCATCACAAGAGAAA	675
3	AAGCCATCACAAGAGAAA	675
4	AAGCCATCACAAGAAAAA	675
5	AAGCCATCACAAGAGAAA	675
6	AAGCCATCACAAGAGAAA	675
7	AAGCCATCACAAGAAAAT	675
8	AAGCCATCACAAAGAAAA	675
9	AAGCCATCACAAAGAAAA	675
10	AAGCCATCACAAAGAAAA	675
11	AAGCCATCACAAAGAAAA	675
12	AAGCCATCACAAGAAAAA	675
13	AAGCCATCACAAGAAAAA	675
14	AAGCCATCACAAGAAAAA	675
15	AAGCCATCACAAGAGAAA	675
16	AAGCCATCACAAGAAAAA	675
17	AAGCCATCACAAGAAAAA	675
18	AAGCCATCACAAAGAGAA	675
19	AAGCCATCACAAAGAAAG	675
20	AAGCCATCACAAAGAAAG	675
21	AAGCCATCACAAAGAAAG	675
22	AAGCCATCACAAAGAAAG	675
23	AAGCCATCACAAAGAAAA	675
24	AAGCCATCACAAAGAAA-	674
25	AAGCCATCACAAAGAAAN	675
26	AAGCCATCACAAAGAAAA	675
27	AAGCCATCACAAAGAAAA	675
28	AAGCCATCACAAAGAAAA	675
29	AAGCCATCACAAGAAAAA	675
30	AAGCCATCACAAGAAAAA	675
31	AAGCCATCACAAAGAAAA	675
32	AAGCCATCACAAGAAAAA	675
33	AAGCCATCACAAGAGAAA	675
34	AAGCCATCACAAGAAAAA	675
35	AAGCCATCACAAGAAAAA	675
36	AAGCCATCACAAAGAAAG	675
37	AAGCCATCACAAAGAAAA	675
38	AAGCCATCACAAGAAAAA	675
39	AAGCCATCACAAGAAAAA	675
40	AAGCCATCACAAGAAAAG	675
41	AAGCCATCACAAGAAAAA	675
42	AAGCCATCACAAAGAAAG	675
43	AAGCCATCACAAAAGAAA	675
44	AAGCCATCACAAGAAAAA	675
45	AAGCCATCACAAAGAAAA	677
46	AAGCCATCACAAAGAAAA	675
47	AAGCCATCACAAAGAAAA	676
48	AAGCCATCACAAGAAAA-	675
49	AAGCCATCACAAAGAAAG	675
50	AACCCAACACAAAAGAAA	675
51	AAGCCATCACAAAGAAAA	675
52	AAGCCATCACAAAGAAAA	675
53	AAGCCATCACAAAGAAAG	675

Sequence Logo: 50% GC base composition

Consensus Threshold: >50%

Colors: 4-color highlighting

Created: 19 Aug 2024

Last Modified: 19 Aug 2024
